# Genome-wide analysis of horizontally acquired genes in the genus *Mycobacterium*

**DOI:** 10.1038/s41598-018-33261-w

**Published:** 2018-10-04

**Authors:** Arup Panda, Michel Drancourt, Tamir Tuller, Pierre Pontarotti

**Affiliations:** 1 Aix-Marseille-Univ., IRD, MEPHI, Institut Hospitalo-Universitaire (IHU) Méditerranée Infection, Marseille, France; 2 Department of Biomedical Engineering, Tel-Aviv University, Ramat Aviv, 69978 Israel; 3 CNRS, Marseille, France

## Abstract

Horizontal gene transfer (HGT) was attributed as a major driving force for the innovation and evolution of prokaryotic genomes. Previously, multiple research endeavors were undertaken to decipher HGT in different bacterial lineages. The genus *Mycobacterium* houses some of the most deadly human pathogens; however, the impact of HGT in *Mycobacterium* has never been addressed in a systematic way. Previous initiatives to explore the genomic imprints of HGTs in *Mycobacterium* were focused on few selected species, specifically among the members of *Mycobacterium tuberculosis* complex. Considering the recent availability of a large number of genomes, the current study was initiated to decipher the probable events of HGTs among 109 completely sequenced *Mycobacterium* species. Our comprehensive phylogenetic analysis with more than 9,000 families of *Mycobacterium* proteins allowed us to list several instances of gene transfers spread across the *Mycobacterium* phylogeny. Moreover, by examining the topology of gene phylogenies here, we identified the species most likely to donate and receive these genes and provided a detailed overview of the putative functions these genes may be involved in. Our study suggested that horizontally acquired foreign genes had played an enduring role in the evolution of *Mycobacterium* genomes and have contributed to their metabolic versatility and pathogenicity.

## Introduction

A significant fraction of genes in all living species was considered to be acquired from genealogically distant species^1–7^. This mode of gene exchange between reproductively isolated species, commonly known as horizontal gene transfer (HGT) or lateral gene transfer, was attributed as a major evolutionary force in several prokaryotic lineages^6,8–10^. Previous studies have implicated horizontally transferred genes in various important traits including novel metabolic pathways^11–13^, oxygenic photosynthesis^14^, antibiotic resistance^15^, pathogenesis^16^ and microbial translation efficiency^17^ and various other features^1–4,6–10^. Moreover, foreign genes were shown to assist microbes in colonizing new niches and in sustaining environmental changes^1–7,18^. Considering their implications in bacterial genome evolution, here in this study, an initiative has been undertaken to trace the probable HGT events among all currently available completely sequenced *Mycobacterium* genomes.

The genus *Mycobacterium* comprises more than 160 species of which about 15 deadly pathogens are commonly encountered in human and other animals^19^. Among the pathogenic *Mycobacterium* species, *M*. *tuberculosis* alone was estimated to have infected one-third of the human population causing more than 2 million annual deaths globally^20^. Pathogenic strains were suggested to originate from their free-living ancestors driven by independent or combined influence of genome reduction, gene duplication, gene rearrangement and HGT evolutionary processes. Horizontal gene transfer, among these, was attributed as a major factor contributing to *Mycobacterium* pathogenesis. Although there are controversies regarding the intensity and extent of HGT among different *Mycobacterium* species, however, it is clear from recent studies that *Mycobacterium* genomes had undergone many episodes of intra and interspecies HGTs acquiring genes from diverse origins including some members of eukaryotic families^21–25^. Along with substantial evidence of HGTs in several *Mycobacterium* genomes, previous studies provided important insights regarding their function and evolutionary importance^21–25^. For instance, foreign genes were shown to play important roles in the evolution of *M*. *ulcerans* (from *M*. *marinum*)^26^, *M*. *avium* subsp. *paratuberculosis*^27^ and in shaping pathogenic potential of *M*. *abscessus*^28^ and *M*. *tuberculosis*^22,23^. However, the contribution of HGT in *Mycobacterium* genome evolution has never been investigated in a systematic way. Earlier studies were mainly focused to find the genomic imprints of HGT in few selected genomes, specifically among the members of *M*. *tuberculosis* complex. Further, those studies were conducted either on selected genomic regions^22,23,25^ or considered only intra-genus gene exchanges between *Mycobacterium* species^24^. To this end, in the present analysis, we considered all available completely sequenced *Mycobacterium* genomes (109 genomes at time of data collection during February 2016) and set-out for a systematic analysis of all probable HGTs that these species may have undergone with any non-*Mycobacterium* species.

To date, various methods have been proposed in order to detect genes acquired through HGT. These approaches can be grouped mainly into two categories (*i*) parametric methods which are based upon atypical sequence composition such as unusual codon usage or GC content and (*ii*) phylogenetic methods which infer HGT by contrasting well-supported gene ancestry with established species phylogeny^1–4,6–8,29–31^. Among these, phylogenetic methods were considered to be superior to any parametric methods^1,7^ and have been widely used in the recent analysis.

To identify the putative HGTs in the selected *Mycobacterium* species, here we searched for phyletic patterns that may indicate potential gene transfer events with non-*Mycobacterium* species in their gene phylogeny. To check the consistency of this approach we employed one computational algorithm (T-REX^32,33^) dedicated for *in-silico* identification of horizontally acquired genes. The main difference between the two approaches is that while phyletic pattern analysis depends on human expertise, T-REX infers probable HGTs based on statistical reconciliation of gene and species trees^32,33^. Considering both these two approaches here we identified several instances of horizontal gene exchanges in *Mycobacterium* genomes which are described in subsequent sections with emphasis on their functional implications. Our comprehensive study suggested that although intra-domain (bacteria) gene exchange is more frequent among *Mycobacterium* genomes, however, *Mycobacterium* species have occasionally received genes from other domains of life. On the functional level, our study suggested that horizontally acquired foreign are integral to several biochemical pathways important for the survival of some *Mycobacterium* as pathogens.

## Materials and Methods

### Collection of dataset and gene clustering with OrthoMCL

To collect a comprehensive dataset, we considered the largest bacterial genome repertoire at NCBI Genebank^34^ (ftp://ftp.ncbi.nlm.nih.gov/genomes/genbank/bacteria/) and retrieved 109 completely sequenced *Mycobacterium* proteomes (Supplementary Table S1). Proteins sharing minimum 30% of amino acid identity over at least 50% of their length were clustered together with the OrthoMCL algorithm using default parameters^35^. OrthoMCL is an all-against-all BLAST search program that cluster homologous sequences (orthologs and recent paralogs) based on Markov gene clustering method^35^. Inflation index is an important parameter that determines the size of the cluster^35^. A lower inflation value (stringent clustering i.e. fewer clusters with more proteins) may place true orthologs in separate clusters, while higher inflation value (lenient clustering i.e. more clusters with fewer proteins) may cluster sequences of different functions together^35^. Here, we considered inflation index value of 1.5 which was suggested to balance sensitivity and specificity while including the maximum number of sequences in the clusters^35^. OrthoMCL resulted in 17,215 families of orthologous proteins (Cluster of Orthologous Groups or COGs) of which 523 groups of short proteins (less than 50 amino acids) and 252 probable species-specific groups containing members from same species (paralogs) were discarded. Remaining 16,440 groups were considered for further analysis.

### Ortholog identification and tree construction

In order to identify probable orthologs outside of *Mycobacterium* genus, the longest member of each remaining COG (16,440) was searched against the NCBI non-redundant (NR) protein database with the BlastP (*v-*2.3.0+) algorithm. For each COG, Blast hits were complemented with other members of the group and filtered with a combination of three cutoffs, minimum e-value 1 × 10^−6^, sequence identity 50% and query coverage 70%. COGs were then classified into two categories based upon the distribution of their significant blast hits, (a) groups showing hits within *Mycobacterium* genus and (b) groups showing hits outside of *Mycobacterium* genus. If a group was found to have multiple significant hits in same species (indicated by same taxonomic identifier), we retained the hit with the highest degree of identity. Here we considered the groups (total 9,014 groups, details in Supplementary File 2) showing at least one significant hit outside of *Mycobacterium* genus and at least 3 significant hits altogether. Amino acid sequences of significant hits were retrieved from NCBI non-redundant (NR) database using Blastdbcmd utility and their full taxonomic lineages were obtained from NCBI taxonomy database using ETE toolkit^36^ with the help of their NCBI Gene Identifier (GI) numbers. For each group, multiple sequence alignment was generated by Muscle (v-3.6) multiple sequence alignment tool under default settings^37^. Phylogenetic trees were constructed in two phases. First, we constructed maximum likelihood trees from each such alignments using double-precision version of FastTree^38^ algorithm with parameters -spr 4, -mlacc 2, –slownni for slower and more exhaustive search and –gamma option which accounts for the uncertainties in rates of evolution at different sites^38^. For each group, the best fitting amino acid substitution model optimized for maximum likelihood trees was detected by ProtTest3^39^ and implemented in FastTree. Currently, FastTree supports three amino acid substation models JTT, WAG, and LG^38^. When ProtTest suggested (for 183 of 9104 groups) any other model we used the best of these three models. For the initial screening here we used FastTree because FastTree was shown to be 100–1,000 times faster than many of other standard maximum likelihood-based phylogenetic tree construction algorithms such as PhyML 3.0 or RAxML^38,40^. However, the maximum likelihood topology of FastTree was suggested to be less accurate than that of RAxML which uses more intensive tree search algorithm^38^. Therefore, to reduce false detection of HGTs all the groups where we found signals for probable HGTs (next section) in FastTree phylogeny were again subjected to phylogenetic tree construction using RAxML^40^ with 100 bootstrap replicates and option for automatic detection of the best substitution model parameters. Finally, HGTs were inferred in RAxML phylogeny when we found similar signals for HGT as in FastTree phylogeny. All these phylogenetic trees have been deposited at http://www.mediterranee-infection.com/article.php?laref=981.

### Detection of HGT by tree-topology analysis

**Figure 1:**
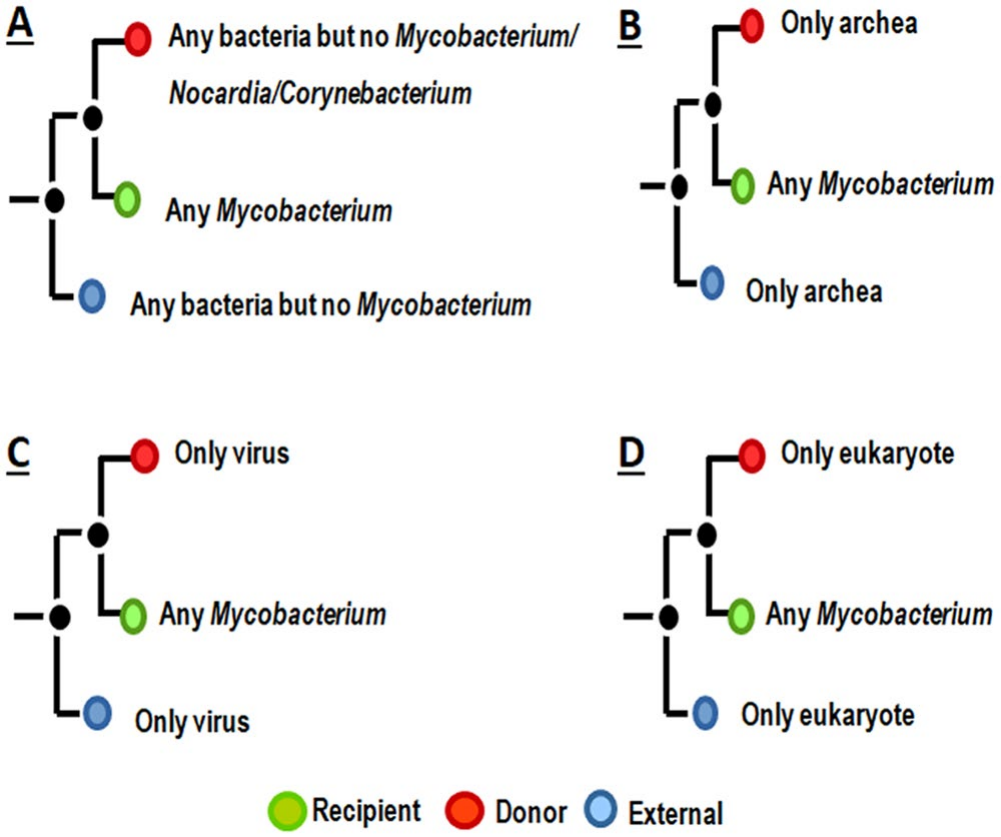
Schematic representation of the phylogenetic patterns that we searched for inferring probable HGTs. Each topology has three basic components: receiver clade (in green), donor clade (in red) and external clade (in blue). The species those we considered for identifying different types of gene transfer are indicated in different panels. (**A**) Gene transfer from other bacteria to *Mycobacterium*, (**B**) gene transfer from archaea to *Mycobacterium*, (**C**) gene transfer from virus to *Mycobacterium* and (**D**) gene transfer from eukaryotes to *Mycobacterium*.

For inferring HGT from tree-topology, we followed the principle as suggested in the previous literature. In a well-supported phylogenetic tree if any gene exhibits higher sequence homology with some distant species rather than with its closest relatives then it is considered as a probable case of HGT^1–4,6–8,29–31^. The pattern that we considered as probable HGT with *Mycobacterium* species is described in Fig. 1. The pattern has basically three components a receiver clade (target *Mycobacterium* groups), a donor clade (the sister group of the receiver clade) and an external clade (the sister group of the donor clade). *Mycobacterium* species were considered to receive genes from other bacterial species, when one or more *Mycobacterium* genes (receiver clade) branched within some unrelated bacteria (with overall minimum bootstrap value 70%) such that there is no *Mycobacterium* gene in up to five neighboring sister clades and there is no member from *Corynebacterium* and *Nocardia* genera (closest relatives of *Mycobacterium*^41^) in the donor clade. To detect gene exchanges with non-bacterial species, we assumed that acquired genes must be present in *Mycobacterium* species but will be absent from other closely related bacterial genomes. Here, we considered similar patterns, however, no bacterial homolog was allowed either in the donor or in the external clade (Fig. 1).

### Inferring donor and receiver clades

Although, our aim was to identify probable HGTs at the species level, however, in most of the cases we found that the donor and receiver groups consisted of genes from multiple genomes. To identify the point of acquisition of foreign genes we reconstructed their ancestral states along the *Mycobacterium* phylogeny using Count^42^, a bioinformatic suite for evolutionary analysis. Given the phyletic distribution of any character along a phylogeny, Count can reconstruct its evolutionary history by various statistical models. Here, the ancestral states were reconstructed through unordered states assumption of Wagner Parsimony model using various gain penalties (1, 3, 5 *etc*.). For *Mycobacterium* species tree needed for this analysis, we first listed all the *Mycobacterium* genomes involved in gene transfer (receiver clade of trees where we found HGT). A representative phylogenetic tree (Supplementary Fig. S1) was constructed for these genomes based on the alignment of 16S rRNA gene sequences retrieved from SILVA (release 128)^43^, a high-quality ribosomal RNA database. Reliable 16S rRNA gene sequences could not be found for few genomes and were not included in the tree. For each probable HGT, a gene presence/absence matrix is generated by mapping the species in receiver groups with the species in the tree and used as the character matrix for ancestral state reconstruction. To identify the evolutionary lineage of donor groups, we searched the lowest taxonomic level (lowest common ancestor) of the members of each donor clade. Donor groups were inferred according to the lowest taxonomic rank (at genus/species/family, *etc*. level) of the common ancestor. Lowest taxonomic rank of the species in donor groups was fetched from ETE toolkit^36^ using their taxonomic identifier.

### Detecting HGT by T-REX algorithm

T-REX is a suite of phylogenetic tools dedicated for several analyses including *in-silico* detection of HGT^32,33^. Given a test and a reference tree, T-REX calculates their proximity by several distance-based measures and predicts minimum-cost scenario HGTs by the progressive reconciliation of those trees. T-REX was optimized by taking into account the evolutionary events including gene duplication and deletion and was shown to be faster and more accurate than most of the other currently available tree discordance methods like LatTrans and RIATA-HGT^32^. The trees generated by the FastTree^38^ algorithm were used as gene trees for this analysis. Species trees were constructed for each gene tree separately. For this, we retrieved the taxonomic ids of all the members of each alignment and fetched their common tree from NCBI taxonomy browser using those ids. Each pair of species and gene tree was then subjected to the T-REX algorithm for inferring probable HGTs. We discarded the “Trivial” (low confidence) HGTs from the prediction and considered the cases where one or more *Mycobacterium* species were listed in the acceptor field with no *Mycobacterium* in the donor field.

### Functional annotations of horizontally transferred genes

Functional annotation of candidate HGTs was done using the InterPro (*v*-66) functional annotation tool^44^. InterPro provides comprehensive information about protein families, domains, and functional sites by integrating signatures from several protein annotation servers including Pfam, PRINTS, PROSITE, SMART, ProDom, SUPERFAMILY, PANTHER, CATH-Gene3D, TIGRFAMs and HAMAP^44^. COGs are clusters of homologous genes and supposed to consist of proteins sharing the same function. Therefore, we choose one representative protein (longest member) from each COG and used it for functional prediction. However, we first tested the utility of our representative protein in retrieving the functional annotation of groups affected by HGT. We retrieved all members of receiver groups for more than 100 families and compared their functional annotations with the functional annotation of the corresponding representative proteins. As expected, we noticed that all the members of each receiver group were composed of similar types of functional domains and were reflected in the composition of the representative proteins. By this way, we could annotate more than 68% of the groups involved in gene transfer with their Gene Ontology (GO) terms and Pfam domains. To get a relative idea, we compared the distribution of Pfam domains and three ontologies (biological process, cellular component, and molecular functions) among the candidate HGTs in reference to their distribution in the whole proteome of 15 *Mycobacterium* species (Supplementary Table S2). Functional annotations of all (76,243) proteins of these 15 *Mycobacterium* species were also retrieved from InterPro^44^. Functional enrichment analysis was performed using Fisher’s exact test with the help of 2 × 2 contingency tables and statistical significance was evaluated through *P*–values. For reliable statistics, functional enrichment analysis was conducted for the GO terms/Pfam domains which appear at least 10 times considering both the dataset (Supplementary File 2). It is noteworthy that since we searched through representative protein, the number of entries in HGT category is based on HGT events rather than genes.

### Mapping of horizontally transferred genes on KEGG pathways

Genes involved in HGT were mapped to the KEGG^45^ (Kyoto Encyclopedia of Genes and Genomes) functional categories using BlastKOALA^46^ genome annotation tool. BlastKOALA assigns KEGG Orthology functional categories (designated by “K” numbers) based upon homology to precompiled databases. Here, the representative sequence (longest) of each HGT COG was searched against “species prokaryotes” database of BlastKOALA using default settings. Enzyme Commission (EC) numbers and pathway information were retrieved following the functional description of the best “K” number assigned to each input sequence. KEGG metabolic pathway map was generated using iPATH Interactive Pathways Explorer (*v*-3)^47^ with the help of ‘K’ numbers.

### Antibiotic resistance and *Mycobacterium* virulence

Candidate HGT proteins were annotated with antibiotic resistance information based upon their sequence similarity with known antibiotic resistance genes. Representative sequences from the candidate HGT COGs were searched against the following databases with BlastP algorithm (*i*) ARDB-Antibiotic Resistance Genes Database (*v*-1.1)^48^, (*ii*) The Comprehensive Antibiotic Resistance Database (CARD) (*v*-1.2.1)^49^, (*iii*) Antibiotic Resistance Gene-ANNOTation database (v-3)^50^, and (*iv*) MEGARes: an Antimicrobial Database for High-Throughput Sequencing (*v*-1.0.1)^51^. These databases are repositories of putative antibiotic resistance genes and related information collected from various resources. Currently, these databases contain 7828, 2311, 1808, and 3824 putative antibiotic resistance gene/protein sequences respectively. To identify the sequences with significant similarity we considered cut-off values of 50% identity over half (50%) of the protein length and e-value less than 10^−5^. HGT proteins were classed according to their putative antibiotic resistance potentiality based upon the functional description of their significant Blast hits. For virulence information we retrieved the protein coding sequences of bacterial virulence factor determinants from the Virulence Factors of Bacterial Pathogens database^52^ and treated in a similar manner. Currently (last updated 24^th^ October 2017), this database contains sequences of 2,595 experimentally verified and 26,524 known and predicted virulence factors. Here, our query sequences were annotated against the experimentally verified dataset.

## Results

### A general overview of HGTs in *Mycobacterium* species

**Figure 2:**
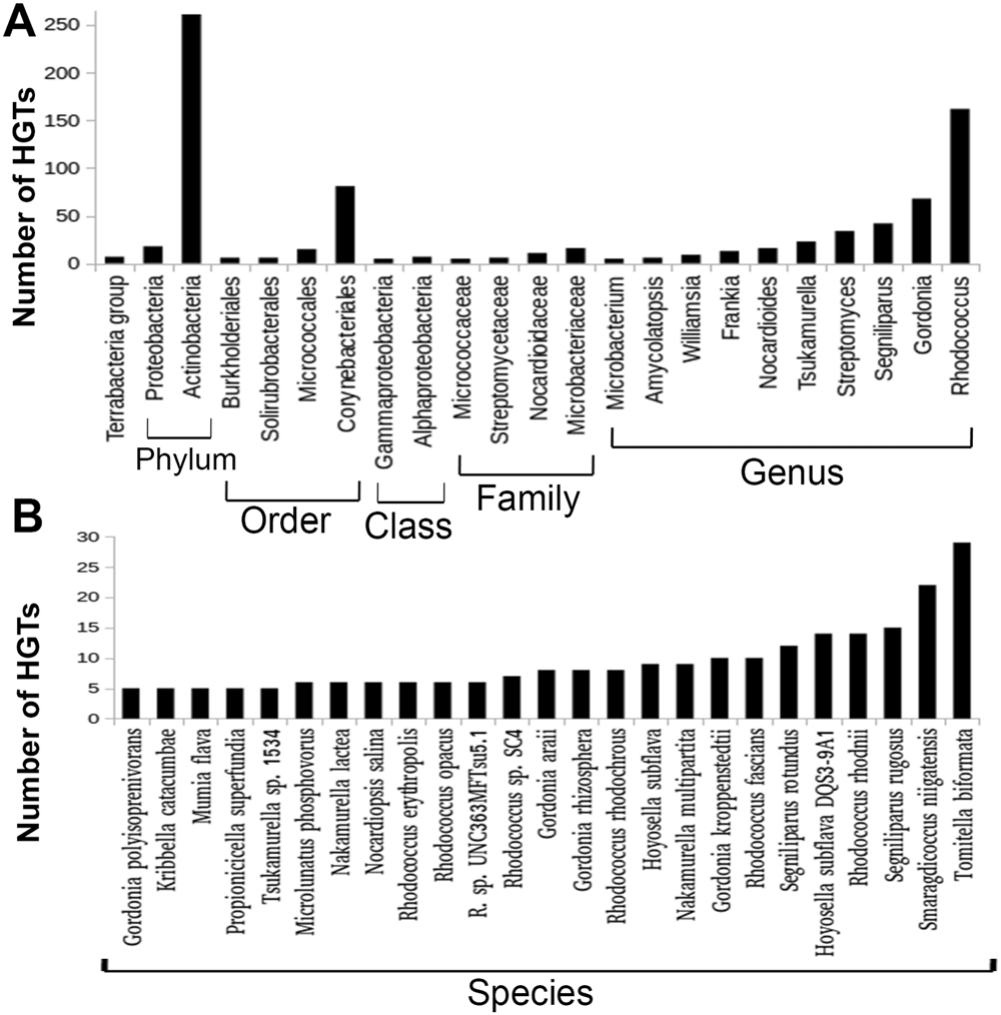
Major donors of *Mycobacterium* foreign genes. This diagram shows the extent of predicted gene transfer events from different bacteria to *Mycobacterium* species (only groups involved in more than 5 HGT events were shown). Candidate HGTs were detected by phylogenetic pattern analysis and donor groups are inferred according to the highest taxonomic rank of species in the donor clades (see main text). Here donor groups are arranged according to their taxonomic rank (**A**) donor bacterial groups at higher taxonomic level and (**B**) donor bacterial groups at species level.

To identify the probable HGTs with other bacterial genomes, we conducted phylogenetic analysis for 9,014 groups showing minimum three unique Blast hits with at least one outside of the *Mycobacterium* genus. To reduce the probability of false detection of HGTs here we followed two phase screening approach. First, we constructed phylogenetic trees for all those groups with FastTree (a relatively faster algorithm) and then the groups showing signals for probable HGTs were further screened with more robust tree construction algorithm RAxML (see material and methods). In our primary screen (FastTree phylogeny) in ~72% (6476/9, 014) of trees we did not find any pattern that could be considered as a probable case of HGT and were discarded. In 5.60% (505/9, 014) of trees HGT pattern was found in multiple branches, suggesting several instances of gene transfer. Because the direction of transfer can’t be detected with confidence we discarded these groups, however considering the possibilities of HGTs a detailed list of these trees with most probable donor and receiver groups are listed in Supplementary File 2. We discarded 29 groups where transfer seems to be due to sequence contamination (Supplementary Table S3). RAxML phylogenetic trees were constructed for remaining 2033 groups where we found clear signals for HGTs in FastTree phylogeny. Considering both RAxmL and FastTree phylogenies, finally we listed 1683 groups where *Mycobacterium* genes seem to be acquired from non-*Mycobacterium* origin (details in Supplementary File 2). We considered these groups as the most probable cases of HGTs and all the subsequent analyses were conducted with these groups. A general inspection suggested that in the majority of these groups transfer has occurred from other bacterial clades to *Mycobacterium*, whereas in 7 groups, we found gene exchange with Viruses (Fig. 2 and Supplementary Table S4). Genes in the receiver clades mostly corresponded to multiple *Mycobacterium* species often distributed in different branches of *Mycobacterium* phylogeny. Our approach of defining donor clades at the lowest taxonomy of the species in donor groups allowed us to detect gene transfer from all the possible taxonomic levels, i.e. from species to species or from higher levels. The major clades that were found to donate genes include the phylum Actinobacteria, Proteobacteria, order Corynebacteriales, Micrococcales and family Streptomycetaceae, Microbacteriaceae. Here, we identified several independent transfers at the species and genus levels. The genera *Rhodococcus*, *Gordonia*, *Segniliparus*, *Streptomyces* and *Tsukamurella* were found to donate genes most frequently while at species level most of the foreign genes were found to come from *Smaragdicoccus niigatensis* DSM 44881 (22 events), *Hoyosella subflava* DQS3-9A1 (14 events), *Tomitella biformata* (29 events), *Rhodococcus fascians* (10 events) and *Segniliparus rotundus* DSM 44985 (12 events). According the characteristics of the recipient *Mycobacterium* genomes we categorized the candidate HGTs into following classes (*i*) where donor group comprised only pathogenic mycobacteria (host associated), (*ii*) donor group comprised only non-pathogenic mycobacteria (*iii*) donor group comprised only opportunistic mycobacteria and (*iv*) HGTs in mixed categories (Supplementary File 2). Here we noticed 37, 69 and 59 independent events of gene transfer to the host-associated, the non-pathogenic and the opportunistic group, respectively. Among these of particular interest may be those that occur during the evolution of the pathogenic group. Our study suggested that most of HGTs in the pathogenic group originated from phylum Actinobacteria (8 events), genus *Rhodococcus* (4 events) and *Streptomyces* (3 events), family Thermomonosporaceae (2 events) and several individual species like *Actinomyces gerencseriae*, *Amycolatopsis methanolica* and *Brevibacterium senegalense* (each 1 event). In 93 COGs at least one significant hit was detected in the viral domain of which our phylogenetic pattern analysis suggested gene transfer in 7 COGs (Supplementary File 2). *Mycobacterium* phage Sbash and *Mycobacterium* phage Whirlwind, a double-stranded DNA virus from the family of Siphoviridae appeared as the common partners. For further phylogenetic assessment, we tested the 1,683 trees where we detected HGT through pattern searching with a tree reconciliation algorithm T-REX (Supplementary File 2 for complete results). 36.72% (618/1,683) of HGTs detected by pattern analysis were also detected by T-REX of which 52.92.5% (327/618) of cases T-REX detected same donor and recipient clades as detected by pattern search (Supplementary Table S5). Although T-REX detected far fewer numbers of probable HGTs, here we noticed an overall similarity in distribution of donor and acceptor groups which suggested a general agreement between the two methods.

### Tracing the evolutionary history of HGTs in *Mycobacterium* species

To identify the point of acquisition of foreign genes we reconstructed the most parsimonious ancestral states of the horizontally acquired genes along the *Mycobacterium* phylogenetic tree. Here, we used Wagner parsimony model (implemented in Count^42^) which infers ancestral states without any restriction on character evolution either in the reversibility of changes or in the number of character transitions (gains or losses). By this way, we could detect the relative time frame of the evolution of horizontally acquired genes. For any group, if candidate HGT genes were suggested (by Count^42^) to be present at the root node of *Mycobacterium* tree with (i.e. lost in some branches and regained in the other branches) or without subsequent gains then they were considered to be most ancestral HGTs acquired by the last common ancestor (LCA) of *Mycobacterium* clade. If candidate HGT genes were suggested to be absent at root node however one or more gains were predicted at internal nodes (with or without any further gain) then they were assumed to be not ancient or not recent (in-between), acquired during the evolution *Mycobacterium* sub-lineages. On the other hand, if gains were predicted only at individual species (affecting only species and no node) then they were assumed to relatively recent HGTs acquired during the evolution of individual species. The results suggested that 641 HGTs were possibly present at the root node of *Mycobacterium* phylogenetic tree (ancient), 678 HGTs were possibly gained at different internal nodes (in-between) and 347 HGTs were gained by different individual *Mycobacterium* species (recent HGTs) (Supplementary File 2). Although relatively fewer numbers of HGTs were mapped as recent HGTs, the results suggested that our candidate HGT genes were gained throughout *Mycobacterium* evolution and the gaining process is likely to be ongoing.

### Functional annotation of genes acquired through HGT

**Figure 3:**
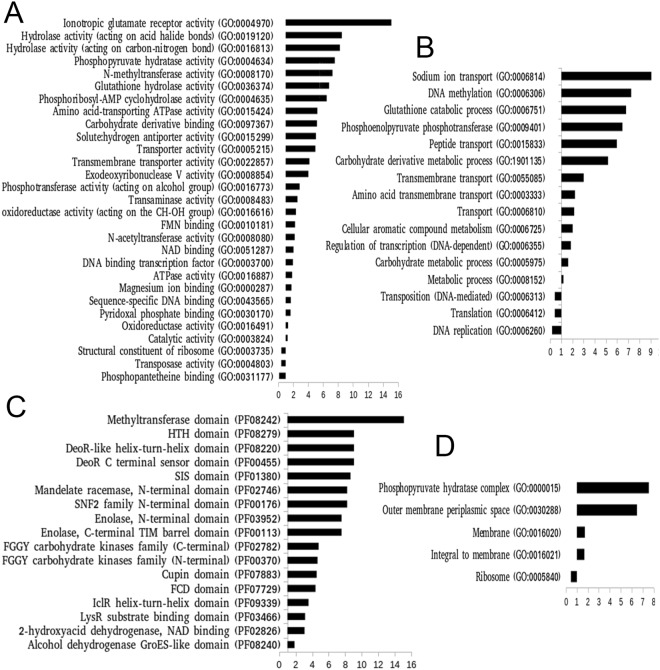
Distribution of three Gene Ontology terms and Pfam domains among candidate HGTs. The diagram shows the relative abundance (odd ratio) of three ontology terms (**A**) Molecular function, (**B**) Biological process, (**D**) Cellular components, and (**C**) Pfam domains among the candidate HGTs in reference to their distribution in all protein-coding genes of 15 *Mycobacterium* species. Here, only the terms showing significant difference in relative abundance between the two groups are shown (*P*-values < 0.05). Significant differences are accessed through Fisher exact test with the null hypothesis that the functional annotations are distributed equally between the two groups (see main text for details).

Using the InterPro protein annotation server, we could assign more than 68% of genes acquired through HGT with their Gene Ontology (GO) terms and protein domain composition. Considering three types of GO ontologies (biological process, cellular component and molecular function) we found 572 unique GO terms among the candidate HGTs and 1512 unique GO terms in the reference set of 15 *Mycobacterium* proteins. When we compared their distribution among these two datasets, most of the GO terms (>90%) were found to occur with similar but at a low frequency. However, we found significant (*P* < 0.05) difference for several functional classes (Supplementary File 2). For instance, biological processes such as methylation (GO:0006306), different types of transport (GO:0006810, GO:0006814, GO:0015833, GO:0055085, GO:0003333), different metabolic processes (GO:0006751, GO:1901135, GO:0006725, GO:0005975, GO:0008152)), *etc*. were found to be significantly (*P* < 0.05) over-represented among the candidate HGTs and DNA replication (GO:0006260), translation (GO:0006412), transposition (GO:0006313) *etc*. were found to be significantly (*P* < 0.05) under-represented (Fig. 3B). Among molecular function ontologies, hydrolase activity (GO:0016813, GO:0019120), ionotropic glutamate receptor activity (GO:0004970), N-methyltransferase activity (GO:0008170) *etc*. were found to be over-represented and terms such as phosphopantetheine binding (GO:0031177), transposase activity (GO:0004803), *etc*. were found to be underrepresented (*P* < 0.05; Fig. 3A). Similarly, phosphopyruvate hydratase complex (GO:0000015), membrane (GO:0016020) *etc*. cellular component ontologies were found to be significantly (*P* < 0.05) over-represented among candidate HGTs with underrepresentation of ribosome (GO:0005840) (Fig. 3D). The vast enrichment of enzymatic activities and transport may be considered as an indication that genes involved in these processes are more frequently exchanged than the genes related to the central machinery of a cell such as DNA replication, translation, *etc*. When we assessed the Pfam domains, we found total 230 unique Pfam domains in candidate HGT dataset and 568 unique Pfam domains in the reference dataset, however only a few of them showed a significant difference in their distribution between these two datasets (Fig. 3C and Supplementary File 2). Some of the Pfam domains that are more likely to undergone gene exchange are DeoR C-terminal sensor domain (PF00455), DeoR-like-helix-turn-helix domain (PF08220), methyltransferase domain (PF08242), sis domain (PF01380), *etc*. Overall these results highlighted the functional characteristics of candidate HGT genes which would help to understand their biological significance.

### Horizontally transferred genes in *Mycobacterium* metabolism

**Figure 4:**
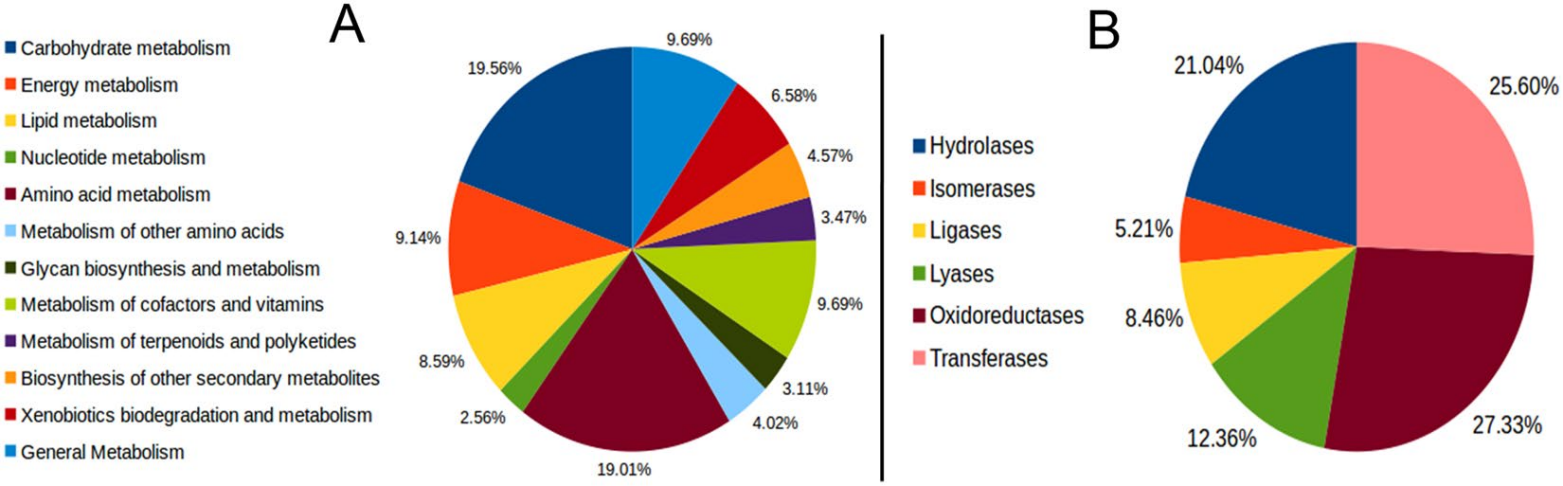
Distribution of different KEGG metabolic categories and enzymatic functions among the candidate HGTs. The diagram shows the distribution of (**A**) different metabolic pathways and (**B**) enzymatic functions among the proteins acquired via HGT events. Metabolic pathways and enzyme commission numbers associated with the candidate HGT proteins were obtained following ‘K’ numbers annotations retrieved from KEGG database. Here, we could retrieve functional annotations for 942 proteins in our dataset.

**Figure 5:**
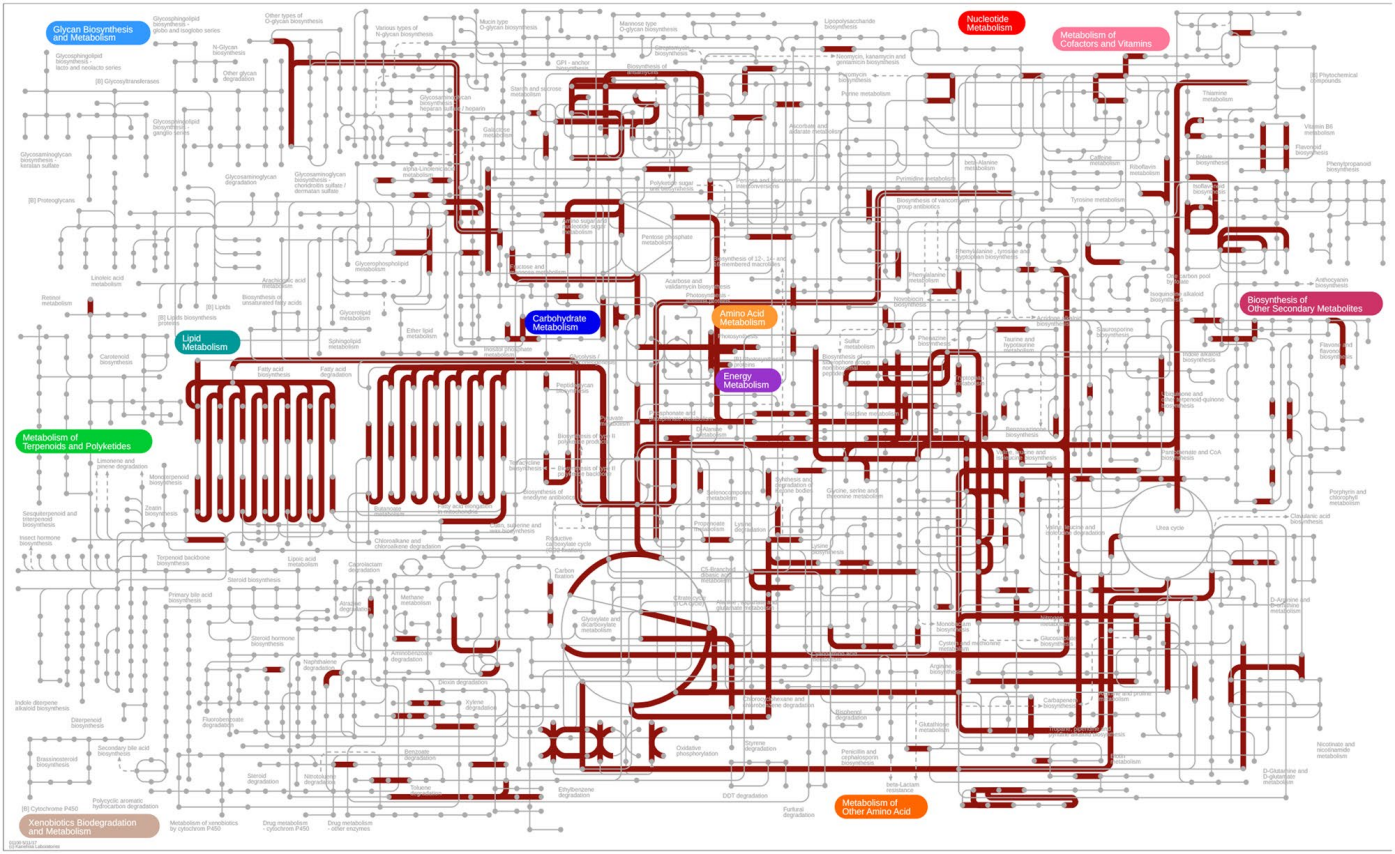
Mapping of candidate HGTs onto the KEGG central metabolic pathways. The figure shows the distribution of candidate HGTs (shown in red thick lines) among the 11 major metabolic pathways in KEGG central metabolic pathways. The 11 major metabolic pathways are color-coded. Candidate HGTs were mapped using the iPath tool^47^ (v-3.0) following KEGG Orthology (KO) annotations as retrieved from KEGG database^45^ (see main text for details).

Previously, a great research endeavor was given to understand whether horizontally acquired genes had played any role in the acquisition of new metabolic traits^11–13^. Considering the general enrichment of several metabolic and enzymatic processes among the candidate HGTs here we decided to explore the issue in more details. When candidate HGTs were annotated against KEGG pathways (see section 2.7), functional annotation could be retrieved for 42% (709 out of 1,683; Supplementary File 2) of HGT COGs of which 34.55% (245/709) were annotated with metabolism (A09100), 16.92% (120/709) with environmental informational processing (A09130), 10.43% (74/709) with genetic information processing (A09120), 18.47% (131/709) with different mixed categories, and 18.19% (129/709) were unclassified (according to KEGG functional hierarchy “A”). When we looked into the metabolic processes in more details (at KEGG functional hierarchy “B”) candidate HGTs were found to participate mostly in carbohydrate metabolism, amino acid metabolism, lipid metabolism, xenobiotic metabolism, energy metabolism, and metabolism of cofactors and vitamins, *etc*. processes (Fig. 4A). A close inspection at KEGG functional hierarchy “C” (compound classification) indicated that a high level of functional specificity exists among the candidate HGTs where 6.9% of annotated genes were found to involve in Butanoate metabolism [PATH:ko00650], 6.10% in propanoate metabolism (PATH:ko00640) and 4.4% in phenylalanine metabolism. Overall these results suggested that candidate HGT genes are likely metabolism genes and are less frequently information storage genes. Horizontally acquired genes were also found to take indispensable part in several catalytic pathways. A larger fraction 57.82% (410/709) of candidate HGTs were mapped to different predicted enzymatic functions (enzyme commission numbers) mostly associated with the catalysis of various biosynthetic pathways. A detailed list of enzymatic activities associated with our candidate HGT genes is provided in Supplementary File 2 however for a general overview we grouped the terms according to their broad classification (first digit) which suggested that 27.33% of candidate HGTs are potential oxidoreductase, 25.59% are transferase, and 21.04% are hydrolase (Fig. 4B).). Finally, to get global overview we mapped the candidate HGTs onto KEGG central metabolic pathways. The map clearly indicated that horizontally acquired genes play indispensable roles in most of the major KEGG metabolic pathways (Fig. 5).

### Horizontally transferred genes invloved in *Mycobacterium* antibiotic resistance and virulence

Horizontally acquired genes were considered to be major contributors to the virulent nature of many pathogens^4,6–8,22–25^. Here, we tested whether the candidate HGT genes had played any role in the origin of antibiotic resistance among *Mycobacterium* species. Considering homology with pre-compiled antibiotic resistance genes from several databases, we found evidence of putative antibiotic resistance like properties in 11 groups out of 1,683 groups with detected gene transfer events (Table 1 and Supplementary File 2). These groups showed homology with genes conferring resistance against drugs as such chloramphenicol, viomycin, aminoglycosides including streptomycin, fluoroquinolones and erythromycin (Table 1). Signatures of their putative antibiotic resistance were further evident from the observations that some of these groups are associated with predicted GO terms related to antibiotic resistance (as predicted by InterPro, Supplementary File 2). When we looked into their origin most of candidate HGTs with signatures of putative antibiotic resistance was found to be acquired from diverse sources including the class Actinobacteria, genus *Amycolatopsis*, *Pseudonocardia* and species *Saccharomonospora cyane* and *Nocardioides luteus*. Further, we found homology with several experimentally verified virulence factor determinants such as mycobactin and urease (Table 1). These factors were known to trigger pathogenic impulse by various mechanisms (Table 1), however, their contribution to the evolution of *Mycobacterium* pathogenicity warrants further experimental verification. Altogether these results highlighted a substantial invasion of several genes with the potential to trigger antibiotic resistance and virulence among *Mycobacterium* species.

**Table 1:** List of candidate HGT genes with putative antibiotic resistance and virulence properties.

HGT COG	Matched from database	Database type	Description
OG_00391	CARD	Antibiotic resistance	resistance to fluoroquinolones
	MEGARes	Antibiotic resistance	Fluoroquinolone-resistant DNA topoisomerases
OG_00412	CARD	Antibiotic resistance	Multi-drug resistance
	MEGARes	Antibiotic resistance	Multi-drug resistance
OG_00519	CARD	Antibiotic resistance	resistance to aminoglycosides
	MEGARes	Antibiotic resistance	Aminoglycoside-resistant
OG_00791	CARD	Antibiotic resistance	confer resistance to para-aminosalicylic acid
OG_00904	MEGARes	Antibiotic resistance	Thiostrepton resistance
OG_00917	CARD	Antibiotic resistance	resistance to mupirocin
OG_01325	VFDB	Virulence factor	controls expression of genes involved in surface remodeling and adaptation to intracellular growth
OG_01462	VFDB	Virulence factor	Cell-association mycobactin participates in iron internalization and/or to serve as a temporary iron-holding molecule.
OG_01490	CARD	Antibiotic resistance	resistance to fluoroquinolones; resistance to pyrazinamide
OG_01562	CARD	Antibiotic resistance	resistance to pyrazinamide
OG_02088	VFDB	Virulence factor	Cell-association mycobactin participates in iron internalization and/or to serve as a temporary iron-holding molecule.
OG_02335	VFDB	Virulence factor	required for intracellular survival and magnesium acquisition
OG_02337	VFDB	Virulence factor	Cell-association mycobactin participates in iron internalization and/or to serve as a temporary iron-holding molecule.
OG_07565	VFDB	Virulence factor	Phenazines biosynthesis
OG_07567	VFDB	Virulence factor	Phenazines biosynthesis
OG_12602	CARD	Antibiotic resistance	confers MLSb phenotype
	MEGARes	Antibiotic resistance	Confers resistance to erythromycin.
OG_15449	CARD	Antibiotic resistance	resistance to mupirocin
OG_16277	MEGARes	Antibiotic resistance	Viomycin_phosphotransferases

Antibiotic and virulence properties are annotated based on homology with known antibiotic resistance genes from various databases (details in main text).

## Discussion

The genus *Mycobacterium* incorporates some of the most deadly human pathogens known to date. Earlier, it has been proposed that extensive genome reduction followed by many episodes of lateral gene transfers had played an enduring role in the evolution of pathogenic *Mycobacterium* strains^23–27,53^. However, the contribution of HGTs in shaping *Mycobacterium* genomes has never been investigated with adequate attention. To this end, here we considered representative proteins from all the currently available completely sequenced *Mycobacterium* genomes and identified the putative cases of HGTs in their phylogenetic trees. To the best of your knowledge, ours is the first effort to quantify gene transfer in mycobacteria through phylogenetic approach at such a large scale. Alternatively, the events of horizontal gene exchange could be detected by surrogate methods based on unrelated genomic signatures or atypical sequence composition^4,6,7,29^. However, these types of signatures were considered to be poor indicators and were shown to be less effective to detect relatively ancient HGTs with very high false positive and false negative detection rates^7,29,30^. Specifically, in the context of *Mycobacterium* species horizontally transferred regions were considered to have similar genomic compositions as the host genomes rendering composition based methods ineffective to provide true estimates of foreign genes^23^. Phylogenetic methods, on the other hand, detect putatively transferred genes considering their similarity with unrelated taxa. The main advantage of phylogenetic methods is that the point of acquisition of the foreign gene as well as the probable donor and recipient lineages can be traced from the tree topology^1,6,7,30^. However, phylogenetic methods are associated with some degree of uncertainty. The efficiency and reliability of phylogenetic approaches largely depend on the breadth and depth of taxon sampling for accessing homology, sequence alignment quality and also upon the choice of model parameters and algorithm for the construction of gene phylogeny^29–31^. Phylogenetic methods are also compromised in their ability to distinguish alternative explanations such as gene duplication, gene loss and sequence contamination which may also mimic true HGTs^7,29,30^. In spite of these disadvantages, when implemented with appropriate model parameters and sufficient taxon sampling phylogenetic methods were suggested to provide highest standard of proof for the identification of HGT^1,7,30^. Considering the advantages of phylogenetic methods, in this study, Maximum likelihood phylogenetic trees were constructed for more than 9,000 *Mycobacterium* protein families and phylogenetic signals indicative of probable horizontal gene exchange were manually searched in the tree topology. Here we considered several measures to minimize the potential errors which may lead to erroneous detection of false positive HGTs. For the initial screening, phylogenetic trees were constructed with FastTree and then each tree where we found signals for HGT were again tested with more sophisticated tree search algorithm RAxML. In both cases we used the best fitting substitution model with optimal algorithm parameters. When a gene branches with sequences from unrelated species with strong statistical support then the probabilities of alternative hypothesis such as gene loss and gene duplication to generate such pattern was suggested to be extremely low such that HGT hypothesis becomes more likely^30^. To reduce such alternative possibilities we considered only those trees (both FastTree and RAxML phylogenies) where mycobacterium genes branched with other bacteria with strong statistical support and without any close relative in the neighboring sister clades. Moreover, we considered the possibility of sequence contamination extensively and filtered-out the groups where our analysis indicated that the pattern may arise due to such artifacts. Finally, we tested the HGTs detected from phylogenetic trees with an algorithm that identifies putative HGTs through statistical reconciliation of gene and species trees. Different methods were suggested to results in non-overlapping sets of transferred genes^29,54^. However, here we noticed a general agreement between the two approaches which suggested that the HGTs that we detected by pattern searching are the highly confident set of *Mycobacterium* horizontally acquired genes.

Previously, a number of initiatives have been undertaken to quantify the extent of HGTs among *Mycobacterium* genomes^21–25,27^. Considering their strict niche specificity initially it was proposed that *Mycobacterium* genomes rarely exchanged their genetic material with any other species^12,27^. *M*. *tuberculosis* was considered clonal, untouched by HGT and evolving only by random genetic drift and selection^55^. However, recent genomic analyses have provided strong evidence for extensive HGTs in and between different *Mycobacterium* species including *M*. *tuberculosis* which raised question about the validity of the clonal paradigm^55^. Considering phylogenetic signals Becq *et al*. and Rosas-Magallanes *et al*. independently speculated that extent of HGTs among *Mycobacterium* species is comparable to any other species^22,23^. However, their studies were limited to few genomic regions from selected *M*. *tuberculosis* strains. Current availability of a large number of completely sequenced *Mycobacterium* genomes has provided us with an opportunity to test their hypothesis in more detail. Our gene phylogeny-based analyses indicated that *Mycobacterium* species have indeed undergone many more events of HGTs than previously anticipated. The topologies of the phylogenetic trees allowed us to identify the species/groups most likely to constitute the donor and recipient clades. To identify the taxonomic lineages of the donor groups we determined the lowest taxonomic level that describes all the members of donor clade. Genomic signature analyses previously suggested three major donor groups, environmental Actinobacteria followed by Proteobacteria and viruses^23^. Here we refined these results in a broader context with more confidence, showing that the phylum Actinobacteria and genera *Rhodococcus*, *Gordonia*, *Streptomyces* and *Tsukamurella* mainly contributed as sources for HGT events. Among the potential donors at the species level, our data suggested that different *Mycobacterium* species have received genes mostly from *Smaragdicoccus niigatensis*, *Hoyosella subflava* DQS3-9A1 and *Tomitella biformata*. Noteworthy, these genera along with *Mycobacterium* mainly comprise environmental organisms residing in soil^56^. Thus our observations agree with the earlier speculation that soil is a major ecosystem for genetic material exchanges between living species.

Along with numerous cases of inter-domain gene exchanges, our analysis indicated a significant fraction of genes has been contributed from other domains of life. Here we noticed several instances of gene exchanges with Viruses. Notably, all the common partners of gene exchange among viruses are *Mycobacterium* phages. Bacteriophages act as a vehicle of gene transfers, however, their identification was considered to be difficult due to rapid evolution^10^. Here we detected traces of phage genes in *Mycobacterium* suggesting their recent evolution.

One important goal of molecular biology studies is to understand the mechanisms by which organisms can exchange their genetic material. Horizontal gene transfer was shown to be mediated by three general mechanisms: transduction, transformation, and conjugation^1–3,7^. *Mycobacterium* genomes are not naturally competent for transformation while there is limited experimental evidence for bacteriophage-mediated transduction^55^. Therefore, mycobacterial species were suggested to be refractory to transduction and transformation but to favor conjugation^23^. Presence of plasmids and phages, the main vehicles of conjugation in different *Mycobacterium* genomes further suggested that conjugation is the most active mechanism of gene transfer in mycobacteria^55^. Based on this evidence here we speculate that a significant fraction of HGTs detected in our analysis has been mediated by conjugative processes.

Given the list of genes that were exchanged between *Mycobacterium* and other bacterial genera, we found it interesting to explore their functional relevance. Thus we noticed that the genes involved in energy production and transformation are more likely to be exchanged than the genes involved in basic housekeeping functions which is in accordance with earlier literature suggesting that genes involved in core biological functions are reluctant to HGT^57^. Specifically, here we noticed a general enrichment of several functional classes such as methylation, and transport among the candidate HGTs. DNA methylation, the only mechanism of epigenetic inherence among prokaryotes is an important component of their gene regulatory system. In the context of mycobacteria, DNA methylation was suggested to promote their persistence under stress full conditions aiding human infection^58^. Transporters are integral to all prokaryotic genomes irrespective of their host association and lifestyle. While their primary purpose is to actively transport different types of metabolites, several types of *Mycobacterium* transporters are implicated in resistance to antibiotics and are used as potential drug targets^59,60^. Although essential for both extra and intracellular lifestyle, *Mycobacterium* genomes encode a comparatively lesser number of transporters than other bacteria such as *Escherichia coli* or *Bacillus subtilis*^60^. To date, there is no clear understanding about their origin, however, the role of HGT in shaping their evolution is suggested by previous studies showing that the exportin Rv0986-Rv0987 (important for the host association of *M*. *tuberculosis)* including several others are acquired foreign genes^22,23^. Considering the general enrichment of different transporters in our HGT dataset here we hypothesized that a significant fraction of *Mycobacterium* transporters have been acquired through HGT.

Metabolic capacity is another important feature with direct impact on bacterial survival. *Mycobacterium* species employ a large fraction of their coding capacity to encode different types of metabolic genes by virtue of which they can synthesize all the amino acids, vitamins, and the enzymes necessary for the production of their primary metabolites^13,61^. It appears from recent studies that interplay of different factors including HGT has contributed to their immense metabolic versatility^12^. *Mycobacterium* genomes were suggested to be shaped by a biphasic evolution of gene acquisition and duplication followed by loss leading to vast expansion of their metabolic genes specifically genes related to lipid metabolism and PE/PPE family^13,53,62^. Here our analysis extends these previous observations showing a wide presence of genes related to different metabolic processes among our candidate HGTs. A significant fraction of candidate HGTs detected in this analysis was mapped to different predictive enzymatic functions broadly belonging to oxidoreductase, transferase and hydrolase categories. Being strict aerobes, mycobacteria are dependent on the extensive use of enzymes related to different oxidative processes required for the generation of ATP. Therefore, mycobacteria are predicted to encode a large number (200) of oxidoreductases, with a significant number of enzymes dedicated for the hydrolysis of ATP and electron transport system^61^. Based on our analysis here we propose that frequent HGTs may have played a key role behind the expansion of their enzyme repository, crucial for the metabolic flexibility which in turn facilitates these bacteria to adapt with different environmental conditions.

Bacterial infections are becoming increasingly difficult to treat due to widespread antibiotic resistance which was co-related with higher frequencies of HGTs among microbial pathogens^15^. HGTs within the microbial community were considered as one of the primary reason for bacterial antibiotic resistance and evolution, maintenance, and transmission of virulence^15^. Due to their thick lipid-rich cell wall, mycobacteria are inherently recalcitrant to several antibiotics^63^. In addition, mycobacteria employ a host of innate and adaptive strategies. Acquired drug resistance in pathogenic *Mycobacterium* species specifically in *M*. *tuberculosis* is suggested to arise from chromosomal mutations rather than through the acquisition of foreign genes^63^. In this study, we noticed putative antibiotic resistance like properties among some of our candidate HGTs. Identification of antibiotic resistance genes among the candidate HGTs will increase our knowledge about their implications in human health and diseases. Despite widespread research, there is little understanding about the molecular mechanisms of *Mycobacterium* pathogenesis. Possible involvement of HGT in *Mycobacterium* virulence has been anticipated in a number of earlier studies^21–24,64^. It is believed that a number of *Mycobacterium* operons with potential virulence activity have originated from the substantial invasion of foreign genes. Here, our detailed gene by gene screen identified several virulence factors among the candidate HGTs suggesting that foreign genes have possibly helped these bacteria to acquire pathogenesis.

## Conclusion

In this work, we reported the first large-scale investigation of putative horizontally acquired genes in the widely diverse genus *Mycobacterium*. Through systematic phylogenetic analysis here we first identified the genes that *Mycobacterium* may have acquired from any non-*Mycobacterium* species then characterize those genes in view of their evolutionary importance. Our observations indicated to their crucial roles in *Mycobacterium* genomes suggesting their potential involvement in different biological functions and metabolic pathways. In addition, our results pointed to some unexplored roles of those genes that have been never been addressed so far. Our computational analyses need biological validations to help to decipher the virulence and resistance mechanisms of pathogenic *Mycobacterium* species and will facilitate the development of new therapies.

## Electronic supplementary material

Supplementary Information

Dataset 1

## Data Availability

The datasets generated and analyzed during the current study are provided in Supplementary File 2. File format: Microsoft excel (.xlsx). Supplementary Tables and Figures associated with this paper are included in Supplementary File 1. File format: Microsoft word (.docx). Phylogenetic trees are deposited at http://www.mediterranee-infection.com/article.php?laref=981.
