# Supplementary material for: Genome-wide analysis of horizontally acquired genes in the genus Mycobacterium
Source: Sci Rep. 2018 Oct 4;8:14817. doi: 10.1038/s41598-018-33261-w (PMC6172269; doi:10.1038/s41598-018-33261-w)
Supplement: Supplementary file 1 — Supplementary Information [file 41598_2018_33261_MOESM1_ESM.docx]

**Additional file1**

**Genome-wide analysis of horizontally acquired genes in the genus *Mycobacterium***

Arup Panda^1,2^, Michel Drancourt^1^, Tamir Tuller^2^, Pierre Pontarotti^1,3^

Affiliation :

1. Aix-Marseille-Univ., IRD, MEPHI, Institut Hospitalo-Universitaire (IHU) Méditerranée Infection, Marseille, France.

2. Department of Biomedical Engineering, Tel-Aviv University, Ramat Aviv 69978, Israel

3. CNRS, Marseille, France.

**email:**

stararup@gmail.com (Arup Panda)

michel.drancourt@univ-amu.fr (Michel Drancourt)

tamirtul@post.tau.ac.il (Tamir Tuller)

pierre.pontarotti@univ-amu.fr (Pierre Pontarotti)

List of contents

| **Contents** | **Page number** |
| --- | --- |
| Table S1: List of genomes considered in this study…………………………………………………… | 2 |
| Table S2: List of genomes used for Pfam functional prediction……………………………………... | 16 |
| Table S3: List of COGs where HGT appeared to be due to contamination ………………………… | 17 |
| Table S4: List of donor groups and number of HGT events identified by pattern searching……… | 21 |
| Table S5: List of donor groups and number of HGT events identified by TRex………………………………. | 34 |
| Figure S1: Mycobacterium phylogenetic tree used for ancestral state reconstruction of candidate HGT genes | 41 |

**Table S1: List of genomes considered in this study.** Genome names, their NCBI accession number, genome size and number of protein coding genes are listed.

| **Name** | **Gene bank**  **Accession** | **Assembly**  **Name** | **Taxon id** | **Species taxon id** | **Characteristics** | **No of proteins** | **Genome size** | **Assembly type** |
| --- | --- | --- | --- | --- | --- | --- | --- | --- |
| Mycobacterium avium subsp. paratuberculosis K-10 | GCA_000007865.1 | ASM786v1 | 262316 | 1764 | high GC; Gram+ | 4350 | 4829781 | Major; Complete; full; latest |
| Mycobacterium tuberculosis CDC1551 | GCA_000008585.1 | ASM858v1 | 83331 | 1773 | high GC; Gram+ | 4189 | 4403837 | Major; Complete; full; latest |
| Mycobacterium bovis BCG str. Pasteur 1173P2 | GCA_000009445.1 | ASM944v1 | 410289 | 1765 | high GC; Gram+ | 3953 | 4374522 | Major; Complete; full; latest |
| Mycobacterium bovis BCG str. Tokyo 172 | GCA_000010685.1 | ASM1068v1 | 561275 | 1765 | high GC; Gram+ | 3950 | 4371711 | Major; Complete; full; latest |
| Mycobacterium ulcerans Agy99 | GCA_000013925.1 | ASM1392v1 | 362242 | 1809 | high GC; Gram+ | 4160 | 5631606 | Major; Complete; full; latest |
| Mycobacterium sp. MCS | GCA_000014165.1 | ASM1416v1 | 164756 | 164756 | high GC; Gram+ | 5615 | 5705448 | Major; Complete; full; latest |
| Mycobacterium avium 104 | GCA_000014985.1 | ASM1498v1 | 243243 | 1764 | high GC; Gram+ | 5120 | 5475491 | Major; Complete; full; latest |
| Mycobacterium smegmatis str. MC2 155 | GCA_000015005.1 | ASM1500v1 | 246196 | 1772 | high GC; Gram+ | 6716 | 6988209 | Major; Complete; full; latest |
| Mycobacterium vanbaalenii PYR-1 | GCA_000015305.1 | ASM1530v1 | 350058 | 110539 | high GC; Gram+ | 5979 | 6491865 | Major; Complete; full; latest |
| Mycobacterium sp. KMS | GCA_000015405.1 | ASM1540v1 | 189918 | 189918 | high GC; Gram+ | 5975 | 5737227 | Major; Complete; full; latest |
| Mycobacterium sp. JLS | GCA_000016005.1 | ASM1600v1 | 164757 | 164757 | high GC; Gram+ | 5739 | 6048425 | Major; Complete; full; latest |
| Mycobacterium tuberculosis H37Ra | GCA_000016145.1 | ASM1614v1 | 419947 | 1773 | high GC; Gram+ | 4034 | 4419977 | Major; Complete; full; latest |
| Mycobacterium gilvum PYR-GCK | GCA_000016365.1 | ASM1636v1 | 350054 | 1804 | high GC; Gram+ | 5579 | 5619607 | Major; Complete; full; latest |
| Mycobacterium tuberculosis F11 | GCA_000016925.1 | ASM1692v1 | 336982 | 1773 | high GC; Gram+ | 3941 | 4424435 | Major; Complete; full; latest |
| Mycobacterium marinum M | GCA_000018345.1 | ASM1834v1 | 216594 | 1781 | high GC; Gram+ | 5452 | 6636827 | Major; Complete; full; latest |
| Mycobacterium tuberculosis KZN 1435 | GCA_000023625.1 | ASM2362v1 | 478434 | 1773 | high GC; Gram+ | 4060 | 4398250 | Major; Complete; full; latest |
| Mycobacterium liflandii 128FXT | GCA_000026445.2 | ASM2644v2 | 459424 | 261524 | high GC; Gram+ | 5064 | 6208955 | Major; Complete; full; latest |
| Mycobacterium leprae Br4923 | GCA_000026685.1 | ASM2668v1 | 561304 | 1769 | high GC; Gram+ | 1604 | 3268071 | Major; Complete; full; latest |
| Mycobacterium abscessus ATCC 19977 | GCA_000069185.1 | ASM6918v1 | 561007 | 36809 | high GC; Gram+ | 4942 | 5067172 | Major; Complete; full; latest |
| Mycobacterium tuberculosis str. Haarlem | GCA_000153685.2 | ASM15368v2 | 395095 | 1773 | high GC; Gram+ | 4036 | 4408224 | Major; Complete; full; latest |
| Mycobacterium tuberculosis KZN 4207 | GCA_000154585.2 | ASM15458v2 | 478433 | 1773 | high GC; Gram+ | 3996 | 4394985 | Major; Complete; full; latest |
| Mycobacterium tuberculosis KZN 605 | GCA_000154605.2 | ASM15460v2 | 478435 | 1773 | high GC; Gram+ | 4002 | 4399120 | Major; Complete; full; latest |
| Mycobacterium kansasii ATCC 12478 | GCA_000157895.2 | ASM15789v2 | 557599 | 1768 | high GC; Gram+ | 5866 | 6432277 | Major; Complete; full; latest |
| Mycobacterium gilvum Spyr1 | GCA_000184435.1 | ASM18443v1 | 278137 | 1804 | high GC; Gram+ | 5349 | 5547747 | Major; Complete; full; latest |
| Mycobacterium tuberculosis W-148 | GCA_000193185.2 | ASM19318v2 | 659019 | 1773 | high GC; Gram+ | 3992 | 4418548 | Major; Complete; full; latest |
| Mycobacterium bovis BCG str. ATCC 35743 | GCA_000194075.3 | ASM19407v3 | 998092 | 1765 | high GC; Gram+ | 4035 | 4334064 | Major; Complete; full; latest |
| Mycobacterium tuberculosis H37Rv | GCA_000195955.2 | ASM19595v2 | 83332 | 1773 | high GC; Gram+ | 4018 | 4411532 | Major; Complete; full; latest |
| Mycobacterium sinense JDM601 | GCA_000214155.1 | ASM21415v1 | 875328 | 875328 | high GC; Gram+ | 4346 | 4643668 | Major; Complete; full; latest |
| Mycobacterium tuberculosis CTRI-2 | GCA_000224435.1 | ASM22443v1 | 707235 | 1773 | high GC; Gram+ | 3946 | 4398525 | Major; Complete; full; latest |
| Mycobacterium rhodesiae NBB3 | GCA_000230895.3 | ASM23089v3 | 710685 | 36814 | high GC; Gram+ | 6147 | 6415739 | Major; Complete; full; latest |
| Mycobacterium bovis BCG str. Mexico | GCA_000234725.1 | ASM23472v1 | 717522 | 1765 | high GC; Gram+ | 3952 | 4350386 | Major; Complete; full; latest |
| Mycobacterium africanum GM041182 | GCA_000253355.1 | ASM25335v1 | 572418 | 33894 | high GC; Gram+ | 3830 | 4389314 | Major; Complete; full; latest |
| Mycobacterium canettii CIPT 140010059 | GCA_000253375.1 | ASM25337v1 | 1048245 | 78331 | high GC; Gram+ | 3861 | 4482059 | Major; Complete; full; latest |
| Mycobacterium sp. MOTT36Y | GCA_000262165.1 | ASM26216v1 | 1168287 | 1168287 | high GC; Gram+ | 5128 | 5613626 | Major; Complete; full; latest |
| Mycobacterium chubuense NBB4 | GCA_000266905.1 | ASM26690v1 | 710421 | 1800 | high GC; Gram+ | 5843 | 5583723 | Major; Complete; full; latest |
| Mycobacterium tuberculosis CCDC5079 | GCA_000270345.1 | ASM27034v1 | 443149 | 1773 | high GC; Gram+ | 3647 | 4398812 | Major; Complete; full; latest |
| Mycobacterium tuberculosis CCDC5180 | GCA_000270365.1 | ASM27036v1 | 443150 | 1773 | high GC; Gram+ | 3591 | 4405981 | Major; Complete; full; latest |
| Mycobacterium paraintracellulare MOTT64 | GCA_000276825.1 | ASM27682v1 | 1138383 | 1138383 | high GC; Gram+ | 5249 | 5501090 | Major; Complete; full; latest |
| Mycobacterium tuberculosis RGTB327 | GCA_000277085.1 | ASM27708v1 | 1091500 | 1773 | high GC; Gram+ | 3691 | 4380119 | Major; Complete; full; latest |
| Mycobacterium tuberculosis RGTB423 | GCA_000277105.1 | ASM27710v1 | 1091501 | 1773 | high GC; Gram+ | 3622 | 4406587 | Major; Complete; full; latest |
| Mycobacterium intracellulare ATCC 13950 | GCA_000277125.1 | ASM27712v1 | 487521 | 1767 | high GC; Gram+ | 5144 | 5402402 | Major; Complete; full; latest |
| Mycobacterium intracellulare MOTT-02 | GCA_000277145.1 | ASM27714v1 | 1138382 | 1767 | high GC; Gram+ | 5149 | 5409696 | Major; Complete; full; latest |
| Mycobacterium tuberculosis H37Rv | GCA_000277735.2 | ASM27773v2 | 83332 | 1773 | high GC; Gram+ | 4036 | 4411709 | Major; Complete; full; latest |
| Mycobacterium abscessus subsp. massiliense str. GO 06 | GCA_000277775.2 | ASM27777v2 | 1198627 | 36809 | high GC; Gram+ | 4558 | 4687873 | Major; Complete; full; latest |
| Mycobacterium smegmatis str. MC2 155 | GCA_000283295.1 | ASM28329v1 | 246196 | 1772 | high GC; Gram+ | 6693 | 6988208 | Major; Complete; full; latest |
| Mycobacterium indicus pranii MTCC 9506 | GCA_000298095.1 | ASM29809v1 | 1232724 | 35617 | high GC; Gram+ | 5254 | 5589007 | Major; Complete; full; latest |
| Mycobacterium neoaurum VKM Ac-1815D | GCA_000317305.3 | ASM31730v3 | 700508 | 1795 | high GC; Gram+ | 4664 | 5421267 | Major; Complete; full; latest |
| Mycobacterium sp. JS623 | GCA_000328565.1 | ASM32856v1 | 212767 | 212767 | high GC; Gram+ | 6939 | 6464916 | Major; Complete; full; latest |
| Mycobacterium tuberculosis 7199-99 | GCA_000331445.1 | ASM33144v1 | 1138877 | 1773 | high GC; Gram+ | 3994 | 4421197 | Major; Complete; full; latest |
| Mycobacterium bovis BCG str. Korea 1168P | GCA_000338715.2 | ASM33871v2 | 1206780 | 1765 | high GC; Gram+ | 4139 | 4376711 | Major; Complete; full; latest |
| Mycobacterium haemophilum DSM 44634 | GCA_000340435.3 | ASM34043v3 | 1202450 | 29311 | high GC; Gram+ | 3728 | 4235765 | Major; Complete; full; latest |
| Mycobacterium tuberculosis str. Erdman = ATCC 35801 | GCA_000350205.1 | ASM35020v1 | 652616 | 1773 | high GC; Gram+ | 4246 | 4392353 | Major; Complete; full; latest |
| Mycobacterium tuberculosis str. Beijing/NITR203 | GCA_000364825.1 | ASM36482v1 | 1306400 | 1773 | high GC; Gram+ | 4110 | 4411128 | Major; Complete; full; latest |
| Mycobacterium tuberculosis str. Haarlem/NITR202 | GCA_000389905.1 | ASM38990v1 | 1304279 | 1773 | high GC; Gram+ | 3681 | 4404786 | Major; Complete; full; latest |
| Mycobacterium tuberculosis CAS/NITR204 | GCA_000389925.1 | ASM38992v1 | 1310114 | 1773 | high GC; Gram+ | 3960 | 4392876 | Major; Complete; full; latest |
| Mycobacterium tuberculosis EAI5/NITR206 | GCA_000389945.1 | ASM38994v1 | 1310115 | 1773 | high GC; Gram+ | 4019 | 4390306 | Major; Complete; full; latest |
| Mycobacterium avium subsp. paratuberculosis MAP4 | GCA_000390085.1 | ASM39008v1 | 1199187 | 1764 | high GC; Gram+ | 4326 | 4829424 | Major; Complete; full; latest |
| Mycobacterium tuberculosis CCDC5079 | GCA_000400615.1 | ASM40061v1 | 443149 | 1773 | high GC; Gram+ | 4156 | 4414325 | Major; Complete; full; latest |
| Mycobacterium sp. VKM Ac-1817D | GCA_000416365.2 | ASM41636v2 | 1273687 | 1273687 | high GC; Gram+ | 6078 | 6324222 | Major; Complete; full; latest |
| Mycobacterium yongonense 05-1390 | GCA_000418535.2 | ASM41853v2 | 1138871 | 1203599 | high GC; Gram+ | 5390 | 5521023 | Major; Complete; full; latest |
| Mycobacterium tuberculosis EAI5 | GCA_000422125.1 | ASM42212v1 | 1306414 | 1773 | high GC; Gram+ | 3902 | 4391174 | Major; Complete; full; latest |
| Mycobacterium abscessus subsp. bolletii 50594 | GCA_000445035.1 | ASM44503v1 | 1303024 | 36809 | high GC; Gram+ | 5185 | 5000473 | Major; Complete; full; latest |
| Mycobacterium abscessus subsp. bolletii CCUG 48898 = JCM 15300 | GCA_000497265.2 | ASM49726v2 | 1001714 | 36809 | high GC; Gram+ | 4950 | 4978382 | Major; Complete; full; latest |
| Mycobacterium tuberculosis HKBS1 | GCA_000572125.1 | ASM57212v1 | 1010834 | 1773 | high GC; Gram+ | 4151 | 4407929 | Major; Complete; full; latest |
| Mycobacterium tuberculosis BT2 | GCA_000572155.1 | ASM57215v1 | 1010835 | 1773 | high GC; Gram+ | 4149 | 4401899 | Major; Complete; full; latest |
| Mycobacterium tuberculosis BT1 | GCA_000572175.1 | ASM57217v1 | 1010836 | 1773 | high GC; Gram+ | 4139 | 4399405 | Major; Complete; full; latest |
| Mycobacterium tuberculosis CCDC5180 | GCA_000572195.1 | ASM57219v1 | 443150 | 1773 | high GC; Gram+ | 4159 | 4414346 | Major; Complete; full; latest |
| Mycobacterium tuberculosis K | GCA_000698475.1 | ASM69847v1 | 1249615 | 1773 | high GC; Gram+ | 4146 | 4385518 | Major; Complete; full; latest |
| Mycobacterium tuberculosis  KIT87190 | GCA_000706665.1 | ASM70666v1 | 1773 | 1773 | high GC; Gram+ | 3912 | 4410788 | Major; Complete; full; latest |
| Mycobacterium marinum E11 | GCA_000723425.2 | E11 | 1131442 | 1781 | high GC; Gram+ | 5308 | 6336293 | Major; Complete; full; latest |
| Mycobacterium tuberculosis  ZMC13-264 | GCA_000738445.1 | ASM73844v1 | 1773 | 1773 | high GC; Gram+ | 3914 | 4411507 | Major; Complete; full; latest |
| Mycobacterium tuberculosis  ZMC13-88 | GCA_000738475.1 | ASM73847v1 | 1773 | 1773 | high GC; Gram+ | 3917 | 4411515 | Major; Complete; full; latest |
| Mycobacterium tuberculosis  96075 | GCA_000756525.1 | ASM75652v1 | 1773 | 1773 | high GC; Gram+ | 3981 | 4379376 | Major; Complete; full; latest |
| Mycobacterium tuberculosis  96121 | GCA_000756545.1 | ASM75654v1 | 1773 | 1773 | high GC; Gram+ | 3994 | 4410945 | Major; Complete; full; latest |
| Mycobacterium abscessus subsp. Bolletii MM1513 | GCA_000758225.1 | ASM75822v1 | 319705 | 36809 | high GC; Gram+ | 4293 | 4501725 | Major; Complete; full; latest |
| Mycobacterium bovis  ATCC BAA-935 | GCA_000758245.1 | ASM75824v1 | 1765 | 1765 | high GC; Gram+ | 3919 | 4358088 | Major; Complete; full; latest |
| Mycobacterium avium subsp. avium 2285 (R) | GCA_000758285.1 | ASM75828v1 | 1299330 | 1764 | high GC; Gram+ | 4618 | 5169415 | Major; Complete; full; latest |
| Mycobacterium abscessus subsp. bolletii 103 | GCA_000758385.1 | ASM75838v1 | 1299325 | 36809 | high GC; Gram+ | 4867 | 5051394 | Major; Complete; full; latest |
| Mycobacterium abscessus subsp. Bolletii MA 1948 | GCA_000758405.1 | ASM75840v1 | 319705 | 36809 | high GC; Gram+ | 4883 | 5064190 | Major; Complete; full; latest |
| Mycobacterium intracellulare 1956 | GCA_000767485.1 | ASM76748v1 | 1299331 | 1767 | high GC; Gram+ | 4664 | 5183048 | Major; Complete; full; latest |
| Mycobacterium smegmatis str. MC2 155 | GCA_000767605.1 | ASM76760v1 | 246196 | 1772 | high GC; Gram+ | 6625 | 6988269 | Major; Complete; full; latest |
| Mycobacterium smegmatis INHR1 | GCA_000767665.1 | ASM76766v1 | 1772 | 1772 | high GC; Gram+ | 6624 | 6988337 | Major; Complete; full; latest |
| Mycobacterium smegmatis INHR2 | GCA_000767705.1 | ASM76770v1 | 1772 | 1772 | high GC; Gram+ | 6623 | 6988302 | Major; Complete; full; latest |
| Mycobacterium abscessus subsp. Bolletii MC1518 | GCA_000770125.1 | ASM77012v1 | 319705 | 36809 | high GC; Gram+ | 4866 | 5049258 | Major; Complete; full; latest |
| Mycobacterium abscessus subsp. abscessus DJO-44274 | GCA_000770175.1 | ASM77017v1 | 1185650 | 36809 | high GC; Gram+ | 4526 | 4686331 | Major; Complete; full; latest |
| Mycobacterium abscessus subsp. abscessus 4529 | GCA_000770215.1 | ASM77021v1 | 1185650 | 36809 | high GC; Gram+ | 4531 | 4687494 | Major; Complete; full; latest |
| Mycobacterium avium subsp. avium DJO-44271 | GCA_000770235.1 | ASM77023v1 | 44454 | 1764 | high GC; Gram+ | 4438 | 5011264 | Major; Complete; full; latest |
| Mycobacterium tuberculosis 49-02 | GCA_000786505.1 | MT49-02 | 1427516 | 1773 | high GC; Gram+ | 4241 | 4412379 | Major; Complete; full; latest |
| Mycobacterium tuberculosis H37RvSiena | GCA_000827085.1 | ASM82708v1 | 1437856 | 1773 | high GC; Gram+ | 3948 | 4410911 | Major; Complete; full; latest |
| Mycobacterium tuberculosis str. Kurono | GCA_000828995.1 | ASM82899v1 | 1445606 | 1773 | high GC; Gram+ | 4340 | 4415078 | Major; Complete; full; latest |
| Mycobacterium avium subsp. hominissuis TH135 | GCA_000829075.1 | ASM82907v1 | 1229671 | 1764 | high GC; Gram+ | 4800 | 4951217 | Major; Complete; full; latest |
| Mycobacterium tuberculosis H37Rv | GCA_000831245.1 | ASM83124v1 | 83332 | 1773 | high GC; Gram+ | 4010 | 4396119 | Major; Complete; full; latest |
| Mycobacterium kansasii 662 | GCA_000831265.1 | ASM83126v1 | 1299326 | 1768 | high GC; Gram+ | 4933 | 6383492 | Major; Complete; full; latest |
| Mycobacterium avium subsp. avium 2285 (S) | GCA_000831285.1 | ASM83128v1 | 1299329 | 1764 | high GC; Gram+ | 4620 | 5197664 | Major; Complete; full; latest |
| Mycobacterium kansasii 824 | GCA_000831305.1 | ASM83130v1 | 1299328 | 1768 | high GC; Gram+ | 4955 | 6402301 | Major; Complete; full; latest |
| Mycobacterium avium subsp. paratuberculosis E1 | GCA_000835225.1 | ASM83522v1 | 1770 | 1764 | high GC; Gram+ | 4140 | 4781002 | Major; Complete; full; latest |
| Mycobacterium avium subsp. paratuberculosis E93 | GCA_000835265.1 | ASM83526v1 | 1770 | 1764 | high GC; Gram+ | 4225 | 4786065 | Major; Complete; full; latest |
| Mycobacterium tuberculosis Beijing-like | GCA_000954155.1 | ASM95415v1 | 1773 | 1773 | high GC; Gram+ | 3829 | 4411216 | Major; Complete; full; latest |
| Mycobacterium bovis BCG str. Moreau RDJ | GCA_000967285.1 | ASM96728v1 | 413996 | 1765 | high GC; Gram+ | 3930 | 4340116 | Major; Complete; full; latest |
| Mycobacterium sp. EPa45 | GCA_001021385.1 | ASM102138v1 | 1545728 | 1545728 | high GC; Gram+ | 5599 | 6177406 | Major; Complete; full; latest |
| Mycobacterium bovis BCG 3281 | GCA_001043255.1 | ASM104325v1 | 33892 | 1765 | high GC; Gram+ | 4186 | 4410431 | Major; Complete; full; latest |
| Mycobacterium abscessus UC22 | GCA_001050395.1 | ASM105039v1 | 1132508 | 36809 | high GC; Gram+ | 5042 | 5257136 | Major; Complete; full; latest |
| Mycobacterium bovis  1595 | GCA_001078615.1 | ASM107861v1 | 1765 | 1765 | high GC; Gram+ | 4358 | 4351712 | Major; Complete; full; latest |
| Mycobacterium goodie X7B | GCA_001187505.1 | ASM118750v1 | 134601 | 134601 | high GC; Gram+ | 6321 | 7105933 | Major; Complete; full; latest |
| Mycobacterium bovis BCG Russia 368 | GCA_001274555.1 | ASM127455v1 | 33892 | 1765 | high GC; Gram+ | 4287 | 4370138 | Major; Complete; full; latest |
| Mycobacterium tuberculosis strain SCAID 187.0 | GCA_001275565.1 | ASM127556v1 | 1773 | 1773 | high GC; Gram+ | 4084 | 4379515 | Major; Complete; full; latest |
| Mycobacterium fortuitum CT6 | GCA_001307545.1 | ASM130754v1 | 1766 | 1766 | high GC; Gram+ | 6020 | 6254616 | Major; Complete; full; latest |
| Mycobacterium abscessus NOV0213 | GCA_001430775.1 | ASM143077v1 | 36809 | 36809 | high GC; Gram+ | 4845 | 5173145 | Major; Complete; full; latest |
| Mycobacterium smegmatis NCTC8159 | GCA_001457595.1 | NCTC8159 | 1772 | 1772 | high GC; Gram+ | 6646 | 6983267 | Major; Complete; full; latest |

**Table S2: List of genomes used for functional enrichment analysis.**

| Serial number | Genome ID | Genome name | No. of proteins |
| --- | --- | --- | --- |
| 1 | GCA_000009445.1 | Mycobacterium bovis BCG str. Pasteur 1173P2 | 3953 |
| 2 | GCA_000013925.1 | Mycobacterium ulcerans agy99 | 4160 |
| 3 | GCA_000014985.1 | Mycobacterium avium 104 | 5120 |
| 4 | GCA_000016145.1 | Mycobacterium tuberculosis H37Ra | 4034 |
| 5 | GCA_000016365.1 | Mycobacterium gilvumycobacterium PYR-GCK | 5579 |
| 6 | GCA_000018345.1 | Mycobacterium marinum M | 5452 |
| 7 | GCA_000069185.1 | Mycobacterium abscessus ATCC 19977 | 4942 |
| 8 | GCA_000157895.2 | Mycobacterium kansasiiATCC 12478 | 5866 |
| 9 | GCA_000253355.1 | Mycobacterium africanum GM041182 | 3830 |
| 10 | GCA_000253375.1 | Mycobacterium canettii CIPT 140010059 | 3861 |
| 11 | GCA_000266905.1 | Mycobacterium chubuense NBB4 | 5843 |
| 12 | GCA_000298095.1 | Mycobacterium indicus pranii MTCC 9506 | 5254 |
| 13 | GCA_000328565.1 | Mycobacterium sp. JS623 | 6939 |
| 14 | GCA_000418535.2 | Mycobacterium yongonense05-1390 | 5390 |
| 15 | GCA_001307545.1 | Mycobacterium fortuitum strain CT6 | 6020 |

**Table S3: List of COGs where HGT appeared to be due to contamination during genome sequencing.** Here we tested wherether the HGTs we identified do indeed belong to the species under study and are not due to contamination during genome sequencing. Contamination of (*a*) target genome (here *Mycobacteria*) with any other genome or (*b*) contamination of any other genome with the target genome both may lead to unusual sequence similarity mimicking true HGT. Contamination of foreign DNA during Mycobacteriun genome sequencing (case a) will results in close homology with that specis rather than any *Mycobacterium* gene. On the other hand, if *Myocobacterium* DNA is incorporated during the sequencing of any other genome (case b) it will show close homology with Mycobacterium genes rather than the native genes. Here we considereed both the cases (*i*) where no close Mycobacterium homolog is found for our target Mycobacterium gene and (*ii*) where no close non-Mycobacterium homolog is found for donor gene (however these cases are not found since our pattern seach will require one or more non-Mycobacterium genes in the external clade). To check possibile contamination, we tested whethere the genes identified as candidate HGTs in case (*i*) are flanked by foreign or native genes. When the candidate HGT genes along with their neighbouring genes in the same genomic scafold showed homology with the donor species rather than with any *Mycobacterium* genes then they were considered to be due to potential contamination. Here we listed the cases where HGT appeared to be due to contamination artifacts. All these groups have been discarded from our main results.

| **COG_ID** | **Taxonomic ids of genomes in recipient**  **group** | **Genomes in recipient**  **Clade** | **Taxonomic ids of genomes in donor**  **group** | **Genomes in donor**  **Clade** |
| --- | --- | --- | --- | --- |
| OG_02275 | 1773 | Mycobacterium tuberculosis  (strain 401416) | 1993 | Actinomadura madurae |
| OG_02780 | 36809 | Mycobacterium abscessus  (strain PAP066) | 1386080 | Bacillus sp. EGD-AK10 |
| OG_04080 | 1773 | Mycobacterium tuberculosis  (strain 401416) | 31962 | Actinomadura rifamycini |
| OG_04255 | 1773 | Mycobacterium tuberculosis  (strain 401416) | 1993 | Actinomadura madurae |
| OG_04585 | 1773 | Mycobacterium tuberculosis  (strain 401416) | 1993 | Actinomadura madurae |
| OG_04759 | 1773 | Mycobacterium tuberculosis  (strain 401416) | 1993 | Actinomadura madurae |
| OG_04915 | 1773 | Mycobacterium tuberculosis  (strain 401416) | 1993 | Actinomadura madurae |
| OG_04969 | 1773 | Mycobacterium tuberculosis  (strain 401416) | 1993 | Actinomadura madurae |
| OG_05005 | 1773 | Mycobacterium tuberculosis  (strain 401416) | 58123 | Spirillospora albida |
| OG_05146 | 1773 | Mycobacterium tuberculosis  (strain 401416) | 1993 | Actinomadura madurae |
| OG_05501 | 1773 | Mycobacterium tuberculosis  (strain 401416) | 31962 | Actinomadura rifamycini |
| OG_06008 | 1773 | Mycobacterium tuberculosis  (strain 401416) | 1993 | Actinomadura madurae |
| OG_06781 | 1773 | Mycobacterium tuberculosis  (strain 401416) | 1993 | Actinomadura madurae |
| OG_06789 | 1773 | Mycobacterium tuberculosis  (strain 401416) | 1993 | Actinomadura madurae |
| OG_07284 | 1773 | Mycobacterium tuberculosis  (strain 401416) | 1993 | Actinomadura madurae |
| OG_08156 | 1773 | Mycobacterium tuberculosis  (strain 401416) | 1993 | Actinomadura madurae |
| OG_08777 | 1773 | Mycobacterium tuberculosis  (strain 401416) | 31962 | Actinomadura rifamycini |
| OG_08995 | 1773 | Mycobacterium tuberculosis  (strain 401416) | 1993 | Actinomadura madurae |
| OG_09544 | 1773 | Mycobacterium tuberculosis  (strain 401416) | 1993 | Actinomadura madurae |
| OG_09647 | 1773 | Mycobacterium tuberculosis  (strain 401416) | 1993 | Actinomadura madurae |
| OG_09795 | 36809 | Mycobacterium abscessus  (strain PAP066) | 1386080 | Bacillus sp. EGD-AK10 |
| OG_09802 | 1773 | Mycobacterium tuberculosis  (strain 401416) | 1993 | Actinomadura madurae |
| OG_10094 | 1773 | Mycobacterium tuberculosis  (strain 401416) | 1993 | Actinomadura madurae |
| OG_10310 | 36809 | Mycobacterium abscessus  (strain PAP066) | 1386080 | Bacillus sp. EGD-AK10 |
| OG_10910 | 1773 | Mycobacterium tuberculosis  (strain 401416) | 1993 | Actinomadura madurae |
| OG_11096 | 1773 | Mycobacterium tuberculosis  (strain 401416) | 1993 | Actinomadura madurae |
| OG_11737 | 1773 | Mycobacterium tuberculosis  (strain 401416) | 1993 | Actinomadura madurae |
| OG_14658 | 1773 | Mycobacterium tuberculosis  (strain 401416) | 1993 | Actinomadura madurae |
| OG_16870 | 1773 | Mycobacterium tuberculosis  (strain 401416) | 58123 | Spirillospora albida |

**Table S4: List of donor groups and number of HGT events identified by pattern searching.** Here we listed all the groups identified as potential donors of *Mycobacterium* foreign genes along with the number of such events. Donor groups are inferred according to the highest taxonomic rank of species in the donor clades (see main text).

| **Donor group name** | **Number of HGT events** | **Taxonomic ID of most recent ancestor** | **Donor group's taxonomic rank** | **Donor group belongs to** |
| --- | --- | --- | --- | --- |
| Alphaproteobacteria, 28211 | 7 | 28211 | class | Bacteria |
| Betaproteobacteria, 28216 | 2 | 28216 | class | Bacteria |
| Deltaproteobacteria, 28221 | 1 | 28221 | class | Bacteria |
| Gammaproteobacteria, 1236 | 5 | 1236 | class | Bacteria |
| Acidobacteriaceae, 204434 | 2 | 204434 | family | Bacteria |
| Bradyrhizobiaceae, 41294 | 2 | 41294 | family | Bacteria |
| Burkholderiaceae, 119060 | 2 | 119060 | family | Bacteria |
| Cellulomonadaceae, 85016 | 1 | 85016 | family | Bacteria |
| Chromatiaceae, 1046 | 1 | 1046 | family | Bacteria |
| Dermacoccaceae, 145357 | 2 | 145357 | family | Bacteria |
| Geodermatophilaceae, 85030 | 4 | 85030 | family | Bacteria |
| Intrasporangiaceae, 85021 | 2 | 85021 | family | Bacteria |
| Microbacteriaceae, 85023 | 16 | 85023 | family | Bacteria |
| Micrococcaceae,1268 | 5 | 1268 | family | Bacteria |
| Micromonosporaceae, 28056 | 3 | 28056 | family | Bacteria |
| Myxococcaceae, 31 | 1 | 31 | family | Bacteria |
| Nocardiaceae, 85025 | 2 | 85025 | family | Bacteria |
| Nocardioidaceae, 85015 | 11 | 85015 | family | Bacteria |
| Propionibacteriaceae, 31957 | 1 | 31957 | family | Bacteria |
| Pseudonocardiaceae, 2070 | 3 | 2070 | family | Bacteria |
| Sphingomonadaceae, 41297 | 4 | 41297 | family | Bacteria |
| Streptomycetaceae, 2062 | 6 | 2062 | family | Bacteria |
| Streptosporangiaceae, 2004 | 1 | 2004 | family | Bacteria |
| Thermomonosporaceae, 2012 | 2 | 2012 | family | Bacteria |
| Acidipropionibacterium, 1912215 | 1 | 1912215 | genus | Bacteria |
| Actinoalloteichus, 65496 | 1 | 65496 | genus | Bacteria |
| Actinomadura, 1988 | 3 | 1988 | genus | Bacteria |
| Actinoplanes, 1865 | 2 | 1865 | genus | Bacteria |
| Aeromicrobium, 2040 | 3 | 2040 | genus | Bacteria |
| Agrococcus, 46352 | 1 | 46352 | genus | Bacteria |
| Amycolatopsis, 1813 | 6 | 1813 | genus | Bacteria |
| Arthrobacter, 1663 | 2 | 1663 | genus | Bacteria |
| Bifidobacterium, 1678 | 2 | 1678 | genus | Bacteria |
| Blastococcus, 38501 | 1 | 38501 | genus | Bacteria |
| Brachybacterium, 43668 | 1 | 43668 | genus | Bacteria |
| Bradyrhizobium, 374 | 1 | 374 | genus | Bacteria |
| Burkholderia, 32008 | 3 | 32008 | genus | Bacteria |
| Candidatus Accumulibacter, 327159 | 1 | 327159 | genus | Bacteria |
| Cellulomonas, 1707 | 2 | 1707 | genus | Bacteria |
| Chelativorans, 449972 | 1 | 449972 | genus | Bacteria |
| Chondromyces, 50 | 1 | 50 | genus | Bacteria |
| Clavibacter, 1573 | 1 | 1573 | genus | Bacteria |
| Dietzia, 37914 | 4 | 37914 | genus | Bacteria |
| Frankia, 1854 | 13 | 1854 | genus | Bacteria |
| Gordonia, 2053 | 68 | 2053 | genus | Bacteria |
| Hyphomicrobium, 81 | 1 | 81 | genus | Bacteria |
| Kibdelosporangium, 2029 | 2 | 2029 | genus | Bacteria |
| Kribbella, 182639 | 1 | 182639 | genus | Bacteria |
| Labrenzia, 478070 | 1 | 478070 | genus | Bacteria |
| Luteipulveratus, 745364 | 3 | 745364 | genus | Bacteria |
| Lysinimicrobium, 1217415 | 1 | 1217415 | genus | Bacteria |
| Microbacterium,33882 | 5 | 33882 | genus | Bacteria |
| Microbispora,2005 | 1 | 2005 | genus | Bacteria |
| Micromonospora, 1873 | 1 | 1873 | genus | Bacteria |
| Modestobacter, 88138 | 1 | 88138 | genus | Bacteria |
| Nocardioides,1839 | 16 | 1839 | genus | Bacteria |
| Nocardiopsis, 2013 | 4 | 2013 | genus | Bacteria |
| Noviherbaspirillum, 1344552 | 1 | 1344552 | genus | Bacteria |
| Novosphingobium, 165696 | 1 | 165696 | genus | Bacteria |
| Oerskovia, 162491 | 1 | 162491 | genus | Bacteria |
| Pandoraea, 93217 | 1 | 93217 | genus | Bacteria |
| Phenylobacterium,20 | 1 | 20 | genus | Bacteria |
| Prauserella, 142577 | 2 | 142577 | genus | Bacteria |
| Pseudomonas, 286 | 1 | 286 | genus | Bacteria |
| Rathayibacter, 33886 | 1 | 33886 | genus | Bacteria |
| Rhodococcus, 1827 | 162 | 1827 | genus | Bacteria |
| Rubrobacter, 42255 | 1 | 42255 | genus | Bacteria |
| Saccharothrix, 2071 | 1 | 2071 | genus | Bacteria |
| Sciscionella, 596495 | 3 | 596495 | genus | Bacteria |
| Segniliparus, 286801 | 42 | 286801 | genus | Bacteria |
| Solirubrobacter, 207599 | 1 | 207599 | genus | Bacteria |
| Sphingomonas, 13687 | 2 | 13687 | genus | Bacteria |
| Streptacidiphilus, 228398 | 2 | 228398 | genus | Bacteria |
| Streptomyces, 1883 | 34 | 1883 | genus | Bacteria |
| Terriglobus, 392733 | 1 | 392733 | genus | Bacteria |
| Thermaerobacter, 73918 | 1 | 73918 | genus | Bacteria |
| Tsukamurella pseudospumae,105579 | 1 | 105579 | genus | Bacteria |
| Tsukamurella, 2060 | 23 | 2060 | genus | Bacteria |
| Williamsia, 85043 | 9 | 85043 | genus | Bacteria |
| Xanthomonas, 338 | 2 | 338 | genus | Bacteria |
| Agrococcus pavilionensis RW1,1330458 | 1 | 1330458 | no rank | Bacteria |
| Burkholderiales Genera incertae sedis,224471 | 1 | 224471 | no rank | Bacteria |
| Cellulomonas carbonis T26,947969 | 3 | 947969 | no rank | Bacteria |
| Cylindrospermum stagnale PCC 7417,56107 | 1 | 56107 | no rank | Bacteria |
| Dietzia cinnamea P4,910954 | 1 | 910954 | no rank | Bacteria |
| Gordonia bronchialis DSM 43247,526226 | 1 | 526226 | no rank | Bacteria |
| Gordonia polyisoprenivorans NBRC 16320 = JCM 10675,1077976 | 1 | 1077976 | no rank | Bacteria |
| Intrasporangium chromatireducens Q5-1,584657 | 4 | 584657 | no rank | Bacteria |
| Prochlorococcus marinus str. MIT 9303,59922 | 1 | 59922 | no rank | Bacteria |
| Rheinheimera nanhaiensis E407-8,562729 | 1 | 562729 | no rank | Bacteria |
| Rhodococcus erythropolis PR4,234621 | 1 | 234621 | no rank | Bacteria |
| Rhodococcus imtechensis RKJ300 = JCM 13270,1165867 | 1 | 1165867 | no rank | Bacteria |
| Saccharothrix espanaensis DSM 44229,1179773 | 2 | 1179773 | no rank | Bacteria |
| Segniliparus rotundus DSM 44985,640132 | 1 | 640132 | no rank | Bacteria |
| Segniliparus rugosus ATCC BAA-974,679197 | 2 | 679197 | no rank | Bacteria |
| Streptomyces afghaniensis 772,1283301 | 1 | 1283301 | no rank | Bacteria |
| Streptomyces auratus AGR0001,1160718 | 1 | 1160718 | no rank | Bacteria |
| Streptomyces zinciresistens K42,700597 | 1 | 700597 | no rank | Bacteria |
| Terrabacteria group,1783272 | 7 | 1783272 | no rank | Bacteria |
| Thermobispora bispora DSM 43833,469371 | 1 | 469371 | no rank | Bacteria |
| unclassified Acidobacteriaceae,112074 | 1 | 112074 | no rank | Bacteria |
| unclassified Betaproteobacteria (miscellaneous),33809 | 1 | 33809 | no rank | Bacteria |
| unclassified Geodermatophilaceae,234661 | 1 | 234661 | no rank | Bacteria |
| Burkholderiales, 80840 | 6 | 80840 | order | Bacteria |
| Catenulisporales, 414714 | 1 | 414714 | order | Bacteria |
| Cellvibrionales, 1706369 | 1 | 1706369 | order | Bacteria |
| Corynebacteriales, 85007 | 81 | 85007 | order | Bacteria |
| Micrococcales, 85006 | 15 | 85006 | order | Bacteria |
| Myxococcales, 29 | 1 | 29 | order | Bacteria |
| Planctomycetales, 112 | 1 | 112 | order | Bacteria |
| Propionibacteriales, 85009 | 2 | 85009 | order | Bacteria |
| Rhizobiales, 356 | 3 | 356 | order | Bacteria |
| Solirubrobacterales, 588673 | 6 | 588673 | order | Bacteria |
| Streptosporangiales, 85012 | 1 | 85012 | order | Bacteria |
| Acidobacteria, 57723 | 1 | 57723 | phylum | Bacteria |
| Actinobacteria,1760 | 261 | 1760 | phylum | Bacteria |
| Cyanobacteria, 1117 | 2 | 1117 | phylum | Bacteria |
| Firmicutes, 1239 | 2 | 1239 | phylum | Bacteria |
| Proteobacteria, 1224 | 18 | 1224 | phylum | Bacteria |
| Acetobacter nitrogenifigens,285268 | 1 | 285268 | species | Bacteria |
| Acidimicrobium ferrooxidans, 53635 | 1 | 53635 | species | Bacteria |
| Acidipropionibacterium acidipropionici, 1748 | 1 | 1748 | species | Bacteria |
| Acidithrix ferrooxidans,1280514 | 3 | 1280514 | species | Bacteria |
| Acidobacterium ailaaui,1382359 | 1 | 1382359 | species | Bacteria |
| Acidovorax sp. Root217,1736492 | 1 | 1736492 | species | Bacteria |
| Actinobacteria bacterium IMCC26207,1641811 | 2 | 1641811 | species | Bacteria |
| Actinobacteria bacterium OV320,1592329 | 1 | 1592329 | species | Bacteria |
| actinobacterium acAMD-5,1504319 | 1 | 1504319 | species | Bacteria |
| Actinocatenispora sera,390989 | 3 | 390989 | species | Bacteria |
| Actinokineospora inagensis,103730 | 1 | 103730 | species | Bacteria |
| Actinokineospora spheciospongiae,909613 | 1 | 909613 | species | Bacteria |
| Actinomadura madurae,1993 | 2 | 1993 | species | Bacteria |
| Actinomyces gerencseriae,52769 | 1 | 52769 | species | Bacteria |
| Actinomyces polynesiensis,1325934 | 1 | 1325934 | species | Bacteria |
| Actinomycetospora chiangmaiensis,402650 | 2 | 402650 | species | Bacteria |
| Actinoplanes rectilineatus,113571 | 1 | 113571 | species | Bacteria |
| Actinoplanes sp. N902-109,649831 | 2 | 649831 | species | Bacteria |
| Actinopolyspora erythraea,414996 | 1 | 414996 | species | Bacteria |
| Aeromicrobium massiliense,1464554 | 4 | 1464554 | species | Bacteria |
| Aeromicrobium sp. Leaf289,1736324 | 1 | 1736324 | species | Bacteria |
| Aeromicrobium sp. Root472D3,1736540 | 1 | 1736540 | species | Bacteria |
| Agreia bicolorata,110935 | 1 | 110935 | species | Bacteria |
| Agrococcus lahaulensis,341722 | 1 | 341722 | species | Bacteria |
| Alloactinosynnema sp. L-07,1653480 | 1 | 1653480 | species | Bacteria |
| Allokutzneria albata,211114 | 1 | 211114 | species | Bacteria |
| Allosalinactinospora lopnorensis,1352348 | 1 | 1352348 | species | Bacteria |
| alpha proteobacterium Mf 1.05b.01,1282876 | 1 | 1282876 | species | Bacteria |
| Amycolatopsis halophila,1069073 | 1 | 1069073 | species | Bacteria |
| Amycolatopsis jejuensis,330084 | 2 | 330084 | species | Bacteria |
| Amycolatopsis methanolica,1814 | 1 | 1814 | species | Bacteria |
| Amycolatopsis nigrescens,381445 | 1 | 381445 | species | Bacteria |
| Amycolatopsis orientalis,31958 | 4 | 31958 | species | Bacteria |
| Amycolatopsis sp. ATCC 39116,385957 | 1 | 385957 | species | Bacteria |
| Amycolatopsis taiwanensis,342230 | 2 | 342230 | species | Bacteria |
| Anaerolineae bacterium SG8_19,1703386 | 2 | 1703386 | species | Bacteria |
| Anaeromyxobacter sp. Fw109-5,404589 | 1 | 404589 | species | Bacteria |
| Arthrobacter sp. 35/47,269454 | 1 | 269454 | species | Bacteria |
| Arthrobacter sp. 35W,1132441 | 1 | 1132441 | species | Bacteria |
| Arthrobacter sp. EpRS66,1743140 | 2 | 1743140 | species | Bacteria |
| Arthrobacter sp. IHBB 11108,1618207 | 2 | 1618207 | species | Bacteria |
| Arthrobacter sp. LS16,1690248 | 1 | 1690248 | species | Bacteria |
| Arthrobacter sp. Soil762,1736401 | 1 | 1736401 | species | Bacteria |
| Arthrobacter sp. Soil764,1736403 | 1 | 1736403 | species | Bacteria |
| Azohydromonas australica,364039 | 1 | 364039 | species | Bacteria |
| Betaproteobacteria bacterium SG8_39,1703390 | 1 | 1703390 | species | Bacteria |
| Beutenbergia cavernae, 84757 | 1 | 84757 | species | Bacteria |
| Blastococcus saxobsidens,138336 | 1 | 138336 | species | Bacteria |
| Blastococcus sp. URHD0036,1380356 | 4 | 1380356 | species | Bacteria |
| Brachybacterium muris, 219301 | 1 | 219301 | species | Bacteria |
| Bradyrhizobium japonicum,375 | 1 | 375 | species | Bacteria |
| Bradyrhizobium sp. LTSPM299,1619233 | 1 | 1619233 | species | Bacteria |
| Brevibacterium album,417948 | 1 | 417948 | species | Bacteria |
| Brevibacterium senegalense,1033736 | 3 | 1033736 | species | Bacteria |
| Brevibacterium sp. VCM10,1381751 | 1 | 1381751 | species | Bacteria |
| Bryobacter aggregatus,360054 | 1 | 360054 | species | Bacteria |
| Caenimonas sp. SL110,1450524 | 1 | 1450524 | species | Bacteria |
| Candidatus Accumulibacter sp. BA-91,1454002 | 1 | 1454002 | species | Bacteria |
| Candidatus Blastococcus massiliensis,1470358 | 2 | 1470358 | species | Bacteria |
| Candidatus Cloacimonas sp. SDB,1732214 | 1 | 1732214 | species | Bacteria |
| Candidatus Entotheonella sp. TSY2,1429439 | 1 | 1429439 | species | Bacteria |
| Candidatus Microthrix parvicella,41950 | 3 | 41950 | species | Bacteria |
| Catelliglobosispora koreensis,129052 | 2 | 129052 | species | Bacteria |
| Caulobacter sp. K31,366602 | 1 | 366602 | species | Bacteria |
| Caulobacteraceae bacterium PMMR1,1382303 | 1 | 1382303 | species | Bacteria |
| Cellulomonas massiliensis,1465811 | 1 | 1465811 | species | Bacteria |
| Cellulomonas sp. A375-1,1672219 | 1 | 1672219 | species | Bacteria |
| Cellulomonas sp. URHE0023,1380354 | 1 | 1380354 | species | Bacteria |
| Cephaloticoccus primus,1548207 | 1 | 1548207 | species | Bacteria |
| Chryseobacterium sp. OV705,1500289 | 1 | 1500289 | species | Bacteria |
| Citricoccus sp. CH26A,1045009 | 1 | 1045009 | species | Bacteria |
| Colwellia psychrerythraea,28229 | 1 | 28229 | species | Bacteria |
| Conexibacter woesei,191495 | 1 | 191495 | species | Bacteria |
| Cupriavidus necator, 106590 | 1 | 106590 | species | Bacteria |
| Cyanobium gracile,59930 | 1 | 59930 | species | Bacteria |
| Cycloclasticus sp. P1,385025 | 1 | 385025 | species | Bacteria |
| Dactylosporangium aurantiacum,35754 | 2 | 35754 | species | Bacteria |
| Demetria terragena,63959 | 2 | 63959 | species | Bacteria |
| Devosia sp. LC5,1502724 | 1 | 1502724 | species | Bacteria |
| Dietzia alimentaria,665550 | 2 | 665550 | species | Bacteria |
| Dietzia sp. UCD-THP,1292020 | 3 | 1292020 | species | Bacteria |
| Endozoicomonas elysicola,305900 | 1 | 305900 | species | Bacteria |
| Ferrimicrobium acidiphilum,121039 | 1 | 121039 | species | Bacteria |
| Frankia elaeagni,222534 | 1 | 222534 | species | Bacteria |
| Frankia inefficax,298654 | 3 | 298654 | species | Bacteria |
| Frankia sp. CN3,298655 | 2 | 298655 | species | Bacteria |
| Frankia sp. Iso899,1283283 | 3 | 1283283 | species | Bacteria |
| Frankia sp. QA3,710111 | 1 | 710111 | species | Bacteria |
| Frondihabitans sp. Leaf304,1736329 | 1 | 1736329 | species | Bacteria |
| Frondihabitans sp. PAMC 28766,1795630 | 1 | 1795630 | species | Bacteria |
| Gallionella capsiferriformans,370405 | 1 | 370405 | species | Bacteria |
| Gemmatirosa kalamazoonesis,861299 | 1 | 861299 | species | Bacteria |
| Geodermatophilaceae bacterium URHB0062,1380353 | 3 | 1380353 | species | Bacteria |
| Georgenia sp. SUBG003,1497974 | 1 | 1497974 | species | Bacteria |
| Gimesia maris, 122 | 1 | 122 | species | Bacteria |
| Glaciibacter superstes,501023 | 3 | 501023 | species | Bacteria |
| Glutamicibacter arilaitensis,256701 | 1 | 256701 | species | Bacteria |
| Glycomyces arizonensis,256035 | 1 | 256035 | species | Bacteria |
| Glycomyces sp. NRRL B-16210,1463821 | 1 | 1463821 | species | Bacteria |
| Glycomyces tenuis,58116 | 1 | 58116 | species | Bacteria |
| Gordonia alkanivorans,84096 | 1 | 84096 | species | Bacteria |
| Gordonia amarae,36821 | 4 | 36821 | species | Bacteria |
| Gordonia araii,263909 | 8 | 263909 | species | Bacteria |
| Gordonia bronchialis,2054 | 4 | 2054 | species | Bacteria |
| Gordonia kroppenstedtii,1121721 | 10 | 1121721 | species | Bacteria |
| Gordonia otitidis,249058 | 1 | 249058 | species | Bacteria |
| Gordonia paraffinivorans,175628 | 2 | 175628 | species | Bacteria |
| Gordonia phthalatica,1136941 | 1 | 1136941 | species | Bacteria |
| Gordonia polyisoprenivorans,84595 | 5 | 84595 | species | Bacteria |
| Gordonia rhizosphera,83341 | 8 | 83341 | species | Bacteria |
| Gordonia rubripertincta,36822 | 4 | 36822 | species | Bacteria |
| Gordonia soli,320799 | 4 | 320799 | species | Bacteria |
| Gordonia sp. HS-NH1,1435068 | 2 | 1435068 | species | Bacteria |
| Gordonia sp. NB4-1Y,1241906 | 1 | 1241906 | species | Bacteria |
| Gordonia sputi,36823 | 2 | 36823 | species | Bacteria |
| Gordonia terrae, 2055 | 1 | 2055 | species | Bacteria |
| Granulicoccus phenolivorans,266854 | 1 | 266854 | species | Bacteria |
| Gryllotalpicola ginsengisoli,444608 | 1 | 444608 | species | Bacteria |
| Gulosibacter molinativorax,256821 | 2 | 256821 | species | Bacteria |
| Herbaspirillum huttiense,863372 | 1 | 863372 | species | Bacteria |
| Herbiconiux sp. YR403,1500280 | 1 | 1500280 | species | Bacteria |
| Herbidospora cretacea,28444 | 1 | 28444 | species | Bacteria |
| Hoyosella subflava DQS3-9A1,443218 | 14 | 443218 | species | Bacteria |
| Hoyosella subflava, 639313 | 9 | 639313 | species | Bacteria |
| Humibacter albus,427754 | 1 | 427754 | species | Bacteria |
| Hydrogenophaga sp. Root209,1736490 | 1 | 1736490 | species | Bacteria |
| Ideonella sakaiensis,1547922 | 1 | 1547922 | species | Bacteria |
| Ilumatobacter coccineus,467094 | 1 | 467094 | species | Bacteria |
| Ilumatobacter nonamiensis,467093 | 2 | 467093 | species | Bacteria |
| Inquilinus limosus,171674 | 1 | 171674 | species | Bacteria |
| Intrasporangium calvum,53358 | 1 | 53358 | species | Bacteria |
| Intrasporangium chromatireducens, 1386088 | 1 | 1386088 | species | Bacteria |
| Janthinobacterium sp. Marseille,375286 | 1 | 375286 | species | Bacteria |
| Jiangella alkaliphila,419479 | 1 | 419479 | species | Bacteria |
| Jiangella gansuensis,281473 | 2 | 281473 | species | Bacteria |
| Jonesia denitrificans,43674 | 1 | 43674 | species | Bacteria |
| Kibdelosporangium aridum,2030 | 3 | 2030 | species | Bacteria |
| Kibdelosporangium sp. MJ126-NF4,703222 | 1 | 703222 | species | Bacteria |
| Kineococcus radiotolerans,131568 | 2 | 131568 | species | Bacteria |
| Kineosporia aurantiaca,49185 | 1 | 49185 | species | Bacteria |
| Kitasatospora mediocidica,58352 | 1 | 58352 | species | Bacteria |
| Knoellia flava,913969 | 1 | 913969 | species | Bacteria |
| Knoellia sinensis,136100 | 1 | 136100 | species | Bacteria |
| Kribbella catacumbae,460086 | 5 | 460086 | species | Bacteria |
| Kribbella flavida, 182640 | 1 | 182640 | species | Bacteria |
| Ktedonobacter racemifer, 363277 | 1 | 363277 | species | Bacteria |
| Kutzneria albida,43357 | 4 | 43357 | species | Bacteria |
| Kutzneria sp. 744,345341 | 1 | 345341 | species | Bacteria |
| Lachnospiraceae bacterium NK4A144,877421 | 1 | 877421 | species | Bacteria |
| Lechevalieria aerocolonigenes,68170 | 1 | 68170 | species | Bacteria |
| Leifsonia rubra,191389 | 1 | 191389 | species | Bacteria |
| Lentzea albidocapillata,40571 | 1 | 40571 | species | Bacteria |
| Longispora albida,203523 | 3 | 203523 | species | Bacteria |
| marine actinobacterium PHSC20C1,312284 | 1 | 312284 | species | Bacteria |
| Marmoricola sp. URHB0036,1298863 | 3 | 1298863 | species | Bacteria |
| Mesorhizobium sp. F7,1574409 | 1 | 1574409 | species | Bacteria |
| Methylocystis sp. SB2,743836 | 1 | 743836 | species | Bacteria |
| Methylocystis sp. SC2,187303 | 1 | 187303 | species | Bacteria |
| Microbacteriaceae bacterium BACL25 MAG-120322-bin65,1655574 | 1 | 1655574 | species | Bacteria |
| Microbacterium gorillae,1231063 | 2 | 1231063 | species | Bacteria |
| Microbacterium mangrovi,1348253 | 2 | 1348253 | species | Bacteria |
| Microbacterium oxydans,82380 | 1 | 82380 | species | Bacteria |
| Microbacterium profundi,450380 | 1 | 450380 | species | Bacteria |
| Microbacterium sp. B19,96765 | 1 | 96765 | species | Bacteria |
| Microbacterium sp. GCS4,1692239 | 1 | 1692239 | species | Bacteria |
| Microbacterium sp. Leaf288,1736323 | 1 | 1736323 | species | Bacteria |
| Microbispora sp. ATCC PTA-5024,316330 | 1 | 316330 | species | Bacteria |
| Microbispora sp. GMKU363,718014 | 2 | 718014 | species | Bacteria |
| Micrococcus luteus,1270 | 1 | 1270 | species | Bacteria |
| Microlunatus phosphovorus,29405 | 6 | 29405 | species | Bacteria |
| Microtetraspora glauca,1996 | 1 | 1996 | species | Bacteria |
| Mumia flava,1348852 | 5 | 1348852 | species | Bacteria |
| Nakamurella lactea,459515 | 6 | 459515 | species | Bacteria |
| Nakamurella multipartita,53461 | 9 | 53461 | species | Bacteria |
| Neisseria sp. 83E34,1692264 | 1 | 1692264 | species | Bacteria |
| Nevskia ramosa,64002 | 1 | 64002 | species | Bacteria |
| Nevskia soli,418856 | 1 | 418856 | species | Bacteria |
| Nitriliruptor alkaliphilus,427918 | 1 | 427918 | species | Bacteria |
| Nitrospira sp. OLB3,1617410 | 1 | 1617410 | species | Bacteria |
| Nocardioides alkalitolerans,281714 | 3 | 281714 | species | Bacteria |
| Nocardioides insulae,394734 | 2 | 394734 | species | Bacteria |
| Nocardioides luteus,1844 | 3 | 1844 | species | Bacteria |
| Nocardioides sp. CF8,110319 | 1 | 110319 | species | Bacteria |
| Nocardioides sp. Iso805N,1283287 | 2 | 1283287 | species | Bacteria |
| Nocardioides sp. J54,935866 | 2 | 935866 | species | Bacteria |
| Nocardioides sp. JS614,196162 | 2 | 196162 | species | Bacteria |
| Nocardioides sp. Root122,1736431 | 3 | 1736431 | species | Bacteria |
| Nocardioides sp. Soil777,1736409 | 1 | 1736409 | species | Bacteria |
| Nocardioides sp. URHA0032,1380388 | 1 | 1380388 | species | Bacteria |
| Nocardiopsis gilva,280236 | 1 | 280236 | species | Bacteria |
| Nocardiopsis salina,245836 | 6 | 245836 | species | Bacteria |
| Nocardiopsis sp. CNT312,1137268 | 1 | 1137268 | species | Bacteria |
| Nonomuraea candida,359159 | 1 | 359159 | species | Bacteria |
| Nonomuraea coxensis,404386 | 1 | 404386 | species | Bacteria |
| Novosphingobium sp. AP12,1144305 | 1 | 1144305 | species | Bacteria |
| Octadecabacter temperatus,1458307 | 1 | 1458307 | species | Bacteria |
| Paenarthrobacter aurescens,43663 | 1 | 43663 | species | Bacteria |
| Paenirhodobacter enshiensis,1105367 | 1 | 1105367 | species | Bacteria |
| Paraglaciecola psychrophila,326544 | 1 | 326544 | species | Bacteria |
| Patulibacter americanus,588672 | 4 | 588672 | species | Bacteria |
| Patulibacter medicamentivorans,1097667 | 3 | 1097667 | species | Bacteria |
| Patulibacter minatonensis,298163 | 2 | 298163 | species | Bacteria |
| Promicromonospora sukumoe,88382 | 2 | 88382 | species | Bacteria |
| Propionicicella superfundia,348582 | 5 | 348582 | species | Bacteria |
| Pseudanabaena sp. PCC 6802,118173 | 1 | 118173 | species | Bacteria |
| Pseudomonas sp. KIE171,159091 | 1 | 159091 | species | Bacteria |
| Rathayibacter sp. Leaf296,1736327 | 2 | 1736327 | species | Bacteria |
| Reyranella massiliensis,445220 | 1 | 445220 | species | Bacteria |
| Rhodococcus aetherivorans,191292 | 4 | 191292 | species | Bacteria |
| Rhodococcus defluvii,1323361 | 2 | 1323361 | species | Bacteria |
| Rhodococcus erythropolis,1833 | 6 | 1833 | species | Bacteria |
| Rhodococcus fascians,1828 | 10 | 1828 | species | Bacteria |
| Rhodococcus hoagii,43767 | 4 | 43767 | species | Bacteria |
| Rhodococcus imtechensis,262776 | 3 | 262776 | species | Bacteria |
| Rhodococcus opacus,37919 | 6 | 37919 | species | Bacteria |
| Rhodococcus pyridinivorans, 103816 | 1 | 103816 | species | Bacteria |
| Rhodococcus rhodnii,38312 | 14 | 38312 | species | Bacteria |
| Rhodococcus rhodochrous,1829 | 8 | 1829 | species | Bacteria |
| Rhodococcus ruber,1830 | 1 | 1830 | species | Bacteria |
| Rhodococcus sp. 114MFTsu3.1,1172184 | 1 | 1172184 | species | Bacteria |
| Rhodococcus sp. AD45,103808 | 1 | 103808 | species | Bacteria |
| Rhodococcus sp. DK17,186196 | 2 | 186196 | species | Bacteria |
| Rhodococcus sp. EsD8,1301088 | 2 | 1301088 | species | Bacteria |
| Rhodococcus sp. JVH1,745408 | 2 | 745408 | species | Bacteria |
| Rhodococcus sp. LB1,1807499 | 2 | 1807499 | species | Bacteria |
| Rhodococcus sp. Leaf278,1736319 | 1 | 1736319 | species | Bacteria |
| Rhodococcus sp. Leaf7,1736210 | 2 | 1736210 | species | Bacteria |
| Rhodococcus sp. MEB064,1587522 | 1 | 1587522 | species | Bacteria |
| Rhodococcus sp. RD6.2,260936 | 1 | 260936 | species | Bacteria |
| Rhodococcus sp. SC4,1793160 | 7 | 1793160 | species | Bacteria |
| Rhodococcus sp. UNC23MFCrub1.1,1449068 | 1 | 1449068 | species | Bacteria |
| Rhodococcus sp. UNC363MFTsu5.1,1449069 | 6 | 1449069 | species | Bacteria |
| Rhodococcus triatomae,300028 | 1 | 300028 | species | Bacteria |
| Rhodococcus wratislaviensis,44752 | 3 | 44752 | species | Bacteria |
| Ruania albidiflava,366586 | 1 | 366586 | species | Bacteria |
| Rudaea cellulosilytica,540746 | 1 | 540746 | species | Bacteria |
| Saccharomonospora sp. CNQ490,1137271 | 1 | 1137271 | species | Bacteria |
| Saccharopolyspora erythraea,1836 | 2 | 1836 | species | Bacteria |
| Saccharopolyspora rectivirgula,28042 | 1 | 28042 | species | Bacteria |
| Saccharopolyspora spinosa,60894 | 2 | 60894 | species | Bacteria |
| Saccharothrix syringae,103733 | 2 | 103733 | species | Bacteria |
| Salinisphaera hydrothermalis, 563188 | 1 | 563188 | species | Bacteria |
| Salinispora pacifica,351187 | 1 | 351187 | species | Bacteria |
| Salisaeta longa,503170 | 1 | 503170 | species | Bacteria |
| Sandaracinus amylolyticus,927083 | 1 | 927083 | species | Bacteria |
| Sciscionella marina,508770 | 3 | 508770 | species | Bacteria |
| Segniliparus rotundus,286802 | 12 | 286802 | species | Bacteria |
| Segniliparus rugosus,286804 | 15 | 286804 | species | Bacteria |
| Serinicoccus marinus,247333 | 1 | 247333 | species | Bacteria |
| Singulisphaera acidiphila, 466153 | 1 | 466153 | species | Bacteria |
| Sinomonas atrocyanea,37927 | 1 | 37927 | species | Bacteria |
| Sinomonas humi,1338436 | 1 | 1338436 | species | Bacteria |
| Smaragdicoccus niigatensis,359359 | 22 | 359359 | species | Bacteria |
| Solirubrobacter soli,363832 | 2 | 363832 | species | Bacteria |
| Sphingomonadales bacterium BRH_c3,1734408 | 1 | 1734408 | species | Bacteria |
| Sphingomonas sp. Leaf357,1736350 | 2 | 1736350 | species | Bacteria |
| Sphingomonas sp. SRS2,133190 | 1 | 133190 | species | Bacteria |
| Sphingomonas sp. YL-JM2C,1336744 | 1 | 1336744 | species | Bacteria |
| Sphingomonas wittichii, 160791 | 1 | 160791 | species | Bacteria |
| Spongiibacter sp. IMCC21906,1620392 | 1 | 1620392 | species | Bacteria |
| Sporichthya polymorpha,35751 | 4 | 35751 | species | Bacteria |
| Stenotrophomonas chelatiphaga,517011 | 1 | 517011 | species | Bacteria |
| Streptacidiphilus jeojiense,436229 | 1 | 436229 | species | Bacteria |
| Streptacidiphilus oryzae,348818 | 1 | 348818 | species | Bacteria |
| Streptomonospora alba,183763 | 1 | 183763 | species | Bacteria |
| Streptomyces atriruber,545121 | 1 | 545121 | species | Bacteria |
| Streptomyces bingchenggensis, 379067 | 1 | 379067 | species | Bacteria |
| Streptomyces hirsutus, 35620 | 1 | 35620 | species | Bacteria |
| Streptomyces hygroscopicus,1912 | 1 | 1912 | species | Bacteria |
| Streptomyces incarnatus,665007 | 1 | 665007 | species | Bacteria |
| Streptomyces iranensis,576784 | 1 | 576784 | species | Bacteria |
| Streptomyces malaysiense,1428626 | 1 | 1428626 | species | Bacteria |
| Streptomyces regalis,68262 | 1 | 68262 | species | Bacteria |
| Streptomyces regensis,68263 | 1 | 68263 | species | Bacteria |
| Streptomyces scabiei,1930 | 1 | 1930 | species | Bacteria |
| Streptomyces scabrisporus,159449 | 1 | 159449 | species | Bacteria |
| Streptomyces sp. AcH 505,352211 | 1 | 352211 | species | Bacteria |
| Streptomyces sp. NBRC 110035,1547867 | 1 | 1547867 | species | Bacteria |
| Streptomyces sp. NRRL F-2664,1463842 | 2 | 1463842 | species | Bacteria |
| Streptomyces sp. NRRL F-3213,1463846 | 1 | 1463846 | species | Bacteria |
| Streptomyces sp. NRRL F-5122,1609098 | 1 | 1609098 | species | Bacteria |
| Streptomyces sp. NRRL F-5123,1463856 | 1 | 1463856 | species | Bacteria |
| Streptomyces sp. NRRL F-5140,1463859 | 2 | 1463859 | species | Bacteria |
| Streptomyces sp. NRRL F-525,1463861 | 1 | 1463861 | species | Bacteria |
| Streptomyces sp. NRRL F-5755,1519475 | 1 | 1519475 | species | Bacteria |
| Streptomyces sp. SBT349,1580539 | 1 | 1580539 | species | Bacteria |
| Streptomyces stelliscabiei,146820 | 1 | 146820 | species | Bacteria |
| Streptomyces vitaminophilus,76728 | 1 | 76728 | species | Bacteria |
| Streptomyces yeochonensis,89050 | 1 | 89050 | species | Bacteria |
| Streptomyces yerevanensis,66378 | 1 | 66378 | species | Bacteria |
| Streptomyces yokosukanensis,67386 | 2 | 67386 | species | Bacteria |
| Streptosporangium amethystogenes,2002 | 1 | 2002 | species | Bacteria |
| Tatumella morbirosei,642227 | 1 | 642227 | species | Bacteria |
| Terrabacter sp. 28,1619947 | 1 | 1619947 | species | Bacteria |
| Terriglobus sp. TAA 43,278961 | 1 | 278961 | species | Bacteria |
| Tetrasphaera japonica,99481 | 3 | 99481 | species | Bacteria |
| Tetrasphaera sp. Soil756,1736399 | 1 | 1736399 | species | Bacteria |
| Thermocrispum municipale,37926 | 1 | 37926 | species | Bacteria |
| Thermomonospora curvata,2020 | 1 | 2020 | species | Bacteria |
| Tomitella biformata,630403 | 29 | 630403 | species | Bacteria |
| Tsukamurella paurometabola,2061 | 2 | 2061 | species | Bacteria |
| Tsukamurella pulmonis,47312 | 3 | 47312 | species | Bacteria |
| Tsukamurella sp. 1534,1151061 | 5 | 1151061 | species | Bacteria |
| Tsukamurella tyrosinosolvens,57704 | 3 | 57704 | species | Bacteria |
| uncultured bacterium WT8,1393214 | 1 | 1393214 | species | Bacteria |
| uncultured bacterium,77133 | 1 | 77133 | species | Bacteria |
| Williamsia sp. D3,1313067 | 1 | 1313067 | species | Bacteria |
| Williamsia sp. Leaf354,1736349 | 1 | 1736349 | species | Bacteria |
| Xanthobacter sp. 126,1131814 | 1 | 1131814 | species | Bacteria |
| Xanthomonas maliensis,1321368 | 1 | 1321368 | species | Bacteria |
| Pseudomonas fluorescens group, species group, 136843 | 1 | 136843 | species group | Bacteria |
| Pseudomonas stutzeri group, species group, 136846 | 1 | 136846 | species group | Bacteria |
| Actinoplanes awajinensis subsp. mycoplanecinus,135947 | 3 | 135947 | subspecies | Bacteria |
| Siphoviridae, 10699 | 5 | 10699 | family | dsDNA viruses |
| Mycobacterium phage Sbash,1567475 | 1 | 1567475 | species | dsDNA viruses |
| Mycobacterium phage Whirlwind,1340826 | 1 | 1340826 | species | dsDNA viruses |

**Table S5: List of donor groups and number of HGT events identified by TRex.** Here we listed all the groups identified as potential donors of *Mycobacterium* foreign genes along with the number of such events. Donor groups are inferred according to the highest taxonomic rank of species in the donor clades (see main text).

| **Donor group name** | **taxonomic id of donor clade** | **Donor group's taxonomic rank** | **Donor group belongs to** | **Number of HGT events** |
| --- | --- | --- | --- | --- |
| Gammaproteobacteria | 1236 | class | Bacteria | 2 |
| Alphaproteobacteria | 28211 | class | Bacteria | 6 |
| Actinobacteria | 1760 | class | Bacteria | 120 |
| Bradyrhizobiaceae | 41294 | family | Bacteria | 1 |
| Intrasporangiaceae | 85021 | family | Bacteria | 1 |
| Myxococcaceae | 31 | family | Bacteria | 1 |
| Oscillospiraceae | 216572 | family | Bacteria | 1 |
| Promicromonosporaceae | 85017 | family | Bacteria | 1 |
| Propionibacteriaceae | 31957 | family | Bacteria | 1 |
| Rhizobiaceae | 82115 | family | Bacteria | 1 |
| Burkholderiaceae | 119060 | family | Bacteria | 2 |
| Cellulomonadaceae | 85016 | family | Bacteria | 2 |
| Dermacoccaceae | 145357 | family | Bacteria | 2 |
| Geodermatophilaceae | 85030 | family | Bacteria | 2 |
| Micromonosporaceae | 28056 | family | Bacteria | 2 |
| Nocardiopsaceae | 83676 | family | Bacteria | 2 |
| Rhodobacteraceae | 31989 | family | Bacteria | 2 |
| Streptomycetaceae | 2062 | family | Bacteria | 2 |
| Micrococcaceae | 1268 | family | Bacteria | 3 |
| Siphoviridae | 10699 | family | Viruses | 3 |
| Nocardiaceae | 85025 | family | Bacteria | 4 |
| Nocardioidaceae | 85015 | family | Bacteria | 7 |
| Pseudonocardiaceae | 2070 | family | Bacteria | 7 |
| Microbacteriaceae | 85023 | family | Bacteria | 8 |
| Actinoalloteichus | 65496 | genus | Bacteria | 1 |
| Actinomadura | 1988 | genus | Bacteria | 1 |
| Amycolatopsis | 1813 | genus | Bacteria | 1 |
| Bacillus | 1386 | genus | Bacteria | 1 |
| Brevibacterium | 1696 | genus | Bacteria | 1 |
| Chelativorans | 449972 | genus | Bacteria | 1 |
| Chondromyces | 50 | genus | Bacteria | 1 |
| Curtobacterium | 2034 | genus | Bacteria | 1 |
| Demequina | 577469 | genus | Bacteria | 1 |
| Devosia | 46913 | genus | Bacteria | 1 |
| Ff47virus | 1920750 | genus | Viruses | 1 |
| Holdemania | 61170 | genus | Bacteria | 1 |
| Hyphomicrobium | 81 | genus | Bacteria | 1 |
| Kribbella | 182639 | genus | Bacteria | 1 |
| Labrenzia | 478070 | genus | Bacteria | 1 |
| Modestobacter | 88138 | genus | Bacteria | 1 |
| Nitrosococcus | 1227 | genus | Bacteria | 1 |
| Nitrososphaera | 497726 | genus | Archaea | 1 |
| Nocardiopsis | 2013 | genus | Bacteria | 1 |
| Novosphingobium | 165696 | genus | Bacteria | 1 |
| Phenylobacterium | 20 | genus | Bacteria | 1 |
| Prauserella | 142577 | genus | Bacteria | 1 |
| Rhodococcus | 1827 | genus | Bacteria | 1 |
| Segniliparus | 286801 | genus | Bacteria | 1 |
| Sulfolobus | 2284 | genus | Archaea | 1 |
| Tetrasphaera | 99479 | genus | Bacteria | 1 |
| Thermaerobacter | 73918 | genus | Bacteria | 1 |
| Xanthomonas | 338 | genus | Bacteria | 1 |
| Actinoplanes | 1865 | genus | Bacteria | 2 |
| Aeromicrobium | 2040 | genus | Bacteria | 2 |
| Luteipulveratus | 745364 | genus | Bacteria | 2 |
| Pseudomonas | 286 | genus | Bacteria | 2 |
| Williamsia | 85043 | genus | Bacteria | 2 |
| Arthrobacter | 1663 | genus | Bacteria | 3 |
| Burkholderia | 32008 | genus | Bacteria | 3 |
| Cellulomonas | 1707 | genus | Bacteria | 3 |
| Nocardioides | 1839 | genus | Bacteria | 3 |
| Tsukamurella | 2060 | genus | Bacteria | 3 |
| Frankia | 1854 | genus | Bacteria | 4 |
| Nocardia | 1817 | genus | Bacteria | 8 |
| Gordonia | 2053 | genus | Bacteria | 11 |
| Streptomyces | 1883 | genus | Bacteria | 17 |
| Rhodococcus | 1827 | genus | Bacteria | 26 |
| Acidimicrobium ferrooxidans DSM 10331 | 525909 | no rank | Bacteria | 1 |
| Actinotalea ferrariae CF5-4 | 948458 | no rank | Bacteria | 1 |
| Alcanivorax pacificus W11-5 | 391936 | no rank | Bacteria | 1 |
| Amycolicicoccus subflavus DQS3-9A1 | 443218 | no rank | Bacteria | 1 |
| Brachybacterium muris UCD-AY4 | 1249481 | no rank | Bacteria | 1 |
| Corynebacterium glyciniphilum AJ 3170 | 1404245 | no rank | Bacteria | 1 |
| Gordonia araii NBRC 100433 | 1073574 | no rank | Bacteria | 1 |
| Gordonia rhizosphera NBRC 16068 | 1108045 | no rank | Bacteria | 1 |
| Gordonia soli NBRC 108243 | 1223545 | no rank | Bacteria | 1 |
| Intrasporangium oryzae NRRL B-24470 | 1386089 | no rank | Bacteria | 1 |
| M phage Bongo | 1088864 | no rank | Viruses | 1 |
| Nakamurella multipartita DSM 44233 | 479431 | no rank | Bacteria | 1 |
| Oidiodendron maius Zn | 913774 | no rank | Eukaryota | 1 |
| Ralstonia eutropha H16 | 381666 | no rank | Bacteria | 1 |
| Rheinheimera nanhaiensis E407-8 | 562729 | no rank | Bacteria | 1 |
| Rhodococcus erythropolis PR4 | 234621 | no rank | Bacteria | 1 |
| Rhodococcus imtechensis RKJ300 | 1165867 | no rank | Bacteria | 1 |
| Saccharothrix espanaensis DSM 44229 | 1179773 | no rank | Bacteria | 1 |
| Salinisphaera hydrothermalis C41B8 | 1304275 | no rank | Bacteria | 1 |
| Salipiger mucosus DSM 16094 | 1123237 | no rank | Bacteria | 1 |
| Segniliparus rotundus DSM 44985 | 640132 | no rank | Bacteria | 1 |
| Streptomyces zinciresistens K42 | 700597 | no rank | Bacteria | 1 |
| Tetrasphaera japonica T1-X7 | 1194083 | no rank | Bacteria | 1 |
| Thioalkalivibrio sulfidiphilus HL-EbGr7 | 396588 | no rank | Bacteria | 1 |
| Gordonia rubripertincta NBRC 101908 | 1077975 | no rank | Bacteria | 2 |
| Rhodococcus rhodnii LMG 5362 | 1273125 | no rank | Bacteria | 2 |
| Myxococcales | 29 | order | Bacteria | 1 |
| Sphingomonadales | 204457 | order | Bacteria | 1 |
| Streptosporangiales | 85012 | order | Bacteria | 1 |
| Rhizobiales | 356 | order | Bacteria | 2 |
| Solirubrobacterales | 588673 | order | Bacteria | 3 |
| Burkholderiales | 80840 | order | Bacteria | 8 |
| Micrococcales | 85006 | order | Bacteria | 8 |
| Corynebacteriales | 85007 | order | Bacteria | 17 |
| Chloroflexi | 200795 | phylum | Bacteria | 1 |
| Cyanobacteria | 1117 | phylum | Bacteria | 1 |
| Proteobacteria | 1224 | phylum | Bacteria | 16 |
| Acetobacter nitrogenifigens | 285268 | species | Bacteria | 1 |
| Actinocatenispora sera | 390989 | species | Bacteria | 1 |
| Actinomadura madurae | 1993 | species | Bacteria | 1 |
| Actinomycetospora chiangmaiensis | 402650 | species | Bacteria | 1 |
| Actinopolyspora halophila | 1850 | species | Bacteria | 1 |
| Actinosporangium NRRL B-3428 | 1463820 | species | Bacteria | 1 |
| Aeromicrobium Root472D3 | 1736540 | species | Bacteria | 1 |
| Amycolatopsis jejuensis | 330084 | species | Bacteria | 1 |
| Amycolatopsis mediterranei | 33910 | species | Bacteria | 1 |
| Amycolatopsis methanolica | 1814 | species | Bacteria | 1 |
| Amycolatopsis nigrescens | 381445 | species | Bacteria | 1 |
| Amycolatopsis orientalis | 31958 | species | Bacteria | 1 |
| Amycolatopsis taiwanensis | 342230 | species | Bacteria | 1 |
| Arthrobacter 161MFSha2.1 | 1151118 | species | Bacteria | 1 |
| Arthrobacter 35W | 1132441 | species | Bacteria | 1 |
| Arthrobacter Soil764 | 1736403 | species | Bacteria | 1 |
| Arthrobacter castelli | 271431 | species | Bacteria | 1 |
| Arthrobacter nitrophenolicus | 683150 | species | Bacteria | 1 |
| Blastococcus URHD0036 | 1380356 | species | Bacteria | 1 |
| Bosea Root483D1 | 1736544 | species | Bacteria | 1 |
| Brachybacterium phenoliresistens | 396014 | species | Bacteria | 1 |
| Bradyrhizobium Ai1a-2 | 196490 | species | Bacteria | 1 |
| Brevibacterium album | 417948 | species | Bacteria | 1 |
| Brevibacterium senegalense | 1033736 | species | Bacteria | 1 |
| Candidatus Entotheonella TSY2 | 1429439 | species | Bacteria | 1 |
| Candidatus Microthrix parvicella | 41950 | species | Bacteria | 1 |
| Caulobacter K31 | 366602 | species | Bacteria | 1 |
| Cephaloticoccus primus | 1548207 | species | Bacteria | 1 |
| Chryseobacterium OV705 | 1500289 | species | Bacteria | 1 |
| Cryptosporangium arvum | 80871 | species | Bacteria | 1 |
| Cupriavidus necator | 106590 | species | Bacteria | 1 |
| Demetria terragena | 63959 | species | Bacteria | 1 |
| Devosia LC5 | 1502724 | species | Bacteria | 1 |
| Francisella TX077308 | 573569 | species | Bacteria | 1 |
| Frankia CN3 | 298655 | species | Bacteria | 1 |
| Frankia EuI1c | 298654 | species | Bacteria | 1 |
| Frankia Iso899 | 1283283 | species | Bacteria | 1 |
| Gallionella capsiferriformans | 370405 | species | Bacteria | 1 |
| Georgenia SUBG003 | 1497974 | species | Bacteria | 1 |
| Glaciibacter superstes | 501023 | species | Bacteria | 1 |
| Glutamicibacter arilaitensis | 256701 | species | Bacteria | 1 |
| Gordonia araii | 263909 | species | Bacteria | 1 |
| Gordonia kroppenstedtii | 1121721 | species | Bacteria | 1 |
| Haliea salexigens | 287487 | species | Bacteria | 1 |
| Hoeflea 108 | 1116369 | species | Bacteria | 1 |
| Hydrogenophaga Root209 | 1736490 | species | Bacteria | 1 |
| Ilumatobacter nonamiensis | 467093 | species | Bacteria | 1 |
| Inquilinus limosus | 171674 | species | Bacteria | 1 |
| Janibacter HTCC2649 | 313589 | species | Bacteria | 1 |
| Jiangella alkaliphila | 419479 | species | Bacteria | 1 |
| Kineococcus radiotolerans | 131568 | species | Bacteria | 1 |
| Kineosphaera limosa | 111564 | species | Bacteria | 1 |
| Kineosporia aurantiaca | 49185 | species | Bacteria | 1 |
| Knoellia flava | 913969 | species | Bacteria | 1 |
| Knoellia sinensis | 136100 | species | Bacteria | 1 |
| Lechevalieria aerocolonigenes | 68170 | species | Bacteria | 1 |
| Leucobacter Ag1 | 1642040 | species | Bacteria | 1 |
| Longispora albida | 203523 | species | Bacteria | 1 |
| M phage Sbash | 1567475 | species | Viruses | 1 |
| Methylocystis SC2 | 187303 | species | Bacteria | 1 |
| Methylogaea oryzae | 1295382 | species | Bacteria | 1 |
| Methylophaga aminisulfidivorans | 230105 | species | Bacteria | 1 |
| Microbacterium mangrovi | 1348253 | species | Bacteria | 1 |
| Microbispora ATCC PTA-5024 | 316330 | species | Bacteria | 1 |
| Nevskia soli | 418856 | species | Bacteria | 1 |
| Nocardia jiangxiensis | 282685 | species | Bacteria | 1 |
| Nocardia seriolae | 37332 | species | Bacteria | 1 |
| Nocardioides JS614 | 196162 | species | Bacteria | 1 |
| Nocardioides Root140 | 1736460 | species | Bacteria | 1 |
| Nocardioides Root190 | 1736488 | species | Bacteria | 1 |
| Nocardioides Soil777 | 1736409 | species | Bacteria | 1 |
| Nocardioides alkalitolerans | 281714 | species | Bacteria | 1 |
| Nocardioides insulae | 394734 | species | Bacteria | 1 |
| Nocardioides luteus | 1844 | species | Bacteria | 1 |
| Nocardiopsis CNT312 | 1137268 | species | Bacteria | 1 |
| Nocardiopsis NRRL B-16309 | 1519494 | species | Bacteria | 1 |
| Nocardiopsis salina | 245836 | species | Bacteria | 1 |
| Nonomuraea candida | 359159 | species | Bacteria | 1 |
| Nonomuraea coxensis | 404386 | species | Bacteria | 1 |
| Novosphingobium AP12 | 1144305 | species | Bacteria | 1 |
| Opitutus terrae | 107709 | species | Bacteria | 1 |
| Paraglaciecola psychrophila | 326544 | species | Bacteria | 1 |
| Patulibacter americanus | 588672 | species | Bacteria | 1 |
| Patulibacter medicamentivorans | 1097667 | species | Bacteria | 1 |
| Patulibacter minatonensis | 298163 | species | Bacteria | 1 |
| Polycyclovorans algicola | 616992 | species | Bacteria | 1 |
| Prauserella rugosa | 43354 | species | Bacteria | 1 |
| Propionibacterium freudenreichii | 1744 | species | Bacteria | 1 |
| Propionicicella superfundia | 348582 | species | Bacteria | 1 |
| Pseudoclavibacter faecalis | 272240 | species | Bacteria | 1 |
| Pseudonocardia AL041005-10 | 445576 | species | Bacteria | 1 |
| Pseudonocardia dioxanivorans | 240495 | species | Bacteria | 1 |
| Rathayibacter Leaf296 | 1736327 | species | Bacteria | 1 |
| Rhodococcus 29MFTsu3.1 | 1150676 | species | Bacteria | 1 |
| Rhodococcus EsD8 | 1301088 | species | Bacteria | 1 |
| Rhodococcus HA99 | 402289 | species | Bacteria | 1 |
| Rhodococcus SC4 | 1793160 | species | Bacteria | 1 |
| Rhodococcus hoagii | 43767 | species | Bacteria | 1 |
| Rhodococcus imtechensis | 262776 | species | Bacteria | 1 |
| Rhodococcus rhodochrous | 1829 | species | Bacteria | 1 |
| Rhodococcus ruber | 1830 | species | Bacteria | 1 |
| Rhodococcus triatomae | 300028 | species | Bacteria | 1 |
| Saccharomonospora CNQ490 | 1137271 | species | Bacteria | 1 |
| Saccharomonospora halophila | 129922 | species | Bacteria | 1 |
| Saccharomonospora marina | 632569 | species | Bacteria | 1 |
| Saccharopolyspora rectivirgula | 28042 | species | Bacteria | 1 |
| Saccharothrix syringae | 103733 | species | Bacteria | 1 |
| Salinarimonas rosea | 552063 | species | Bacteria | 1 |
| Segniliparus rugosus | 286804 | species | Bacteria | 1 |
| Solirubrobacter soli | 363832 | species | Bacteria | 1 |
| Sorangium cellulosum | 56 | species | Bacteria | 1 |
| Stenotrophomonas chelatiphaga | 517011 | species | Bacteria | 1 |
| Steroidobacter denitrificans | 465721 | species | Bacteria | 1 |
| Streptacidiphilus oryzae | 348818 | species | Bacteria | 1 |
| Streptomonospora alba | 183763 | species | Bacteria | 1 |
| Streptomyces CNQ329 | 1298879 | species | Bacteria | 1 |
| Streptomyces MUSC136T | 1428626 | species | Bacteria | 1 |
| Streptomyces NRRL F-2664 | 1463842 | species | Bacteria | 1 |
| Streptomyces NRRL WC-3618 | 1519490 | species | Bacteria | 1 |
| Streptomyces Tu 6176 | 1470557 | species | Bacteria | 1 |
| Streptomyces clavuligerus | 1901 | species | Bacteria | 1 |
| Streptomyces diastatochromogenes | 42236 | species | Bacteria | 1 |
| Streptomyces hygroscopicus | 1912 | species | Bacteria | 1 |
| Streptomyces incarnatus | 665007 | species | Bacteria | 1 |
| Streptomyces iranensis | 576784 | species | Bacteria | 1 |
| Streptomyces pseudovenezuelae | 67350 | species | Bacteria | 1 |
| Streptomyces scabiei | 1930 | species | Bacteria | 1 |
| Streptomyces scabrisporus | 159449 | species | Bacteria | 1 |
| Streptomyces yokosukanensis | 67386 | species | Bacteria | 1 |
| Tsukamurella pulmonis | 47312 | species | Bacteria | 1 |
| Tsukamurella tyrosinosolvens | 57704 | species | Bacteria | 1 |
| Aeromicrobium massiliense | 1464554 | species | Bacteria | 2 |
| Arthrobacter EpRS66 | 1743140 | species | Bacteria | 2 |
| Gordonia rubripertincta | 36822 | species | Bacteria | 2 |
| Jiangella gansuensis | 281473 | species | Bacteria | 2 |
| Kutzneria albida | 43357 | species | Bacteria | 2 |
| Microbacterium G3 | 1231063 | species | Bacteria | 2 |
| Nakamurella lactea | 459515 | species | Bacteria | 2 |
| Nakamurella multipartita | 53461 | species | Bacteria | 2 |
| Rhodococcus DK17 | 186196 | species | Bacteria | 2 |
| Rhodococcus equi | 43767 | species | Bacteria | 2 |
| Rhodococcus fascians | 1828 | species | Bacteria | 2 |
| Segniliparus rotundus | 286802 | species | Bacteria | 2 |
| Streptomyces sulphureus | 47758 | species | Bacteria | 2 |
| Terrabacter 28 | 1619947 | species | Bacteria | 2 |
| Tetrasphaera japonica | 99481 | species | Bacteria | 2 |
| Gordonia polyisoprenivorans | 84595 | species | Bacteria | 3 |
| Marmoricola URHB0036 | 1298863 | species | Bacteria | 3 |
| Rhodococcus erythropolis | 1833 | species | Bacteria | 3 |
| Rhodococcus rhodnii | 38312 | species | Bacteria | 3 |
| Saccharopolyspora spinosa | 60894 | species | Bacteria | 3 |
| Williamsia D3 | 1313067 | species | Bacteria | 3 |
| Kribbella catacumbae | 460086 | species | Bacteria | 4 |
| Smaragdicoccus niigatensis | 359359 | species | Bacteria | 5 |
| Cystobacterineae | 80811 | suborder | Bacteria | 1 |
| Actinoplanes awajinensis | 135947 | subspecies | Bacteria | 1 |

**Figure S1: Mycobacterium phylogenetic tree used for ancestral state reconstruction of candidate HGT genes.** The tree is constructed based on alignment of 16S RNA sequences using Fasttree.
